# A Rational Optimization Approach for the Development of a Multiplexed Lateral Flow Immunoassay: Detection of Nonepithelial Ovarian Cancer Markers in Human Serum

**DOI:** 10.1002/advs.202523192

**Published:** 2026-02-25

**Authors:** Aida Abdelwahed, Carl E. Eskildsen, Luca Panariello, André Shamsabadi, Christy J. Sadler, Edmund H. Wilkes, Federico Galvanin, Yuxi Cheng, Sara Carvalho, Srdjan Saso, Molly M. Stevens

**Affiliations:** ^1^ Department of Materials Depart of Bioengineering and Institute of Biomedical Engineering Imperial College London London UK; ^2^ Department of Physiology Anatomy and Genetics Department of Engineering Science and Kavli Institute For Nanoscience Discovery University of Oxford Oxford UK; ^3^ Department of Medical Biochemistry and Biophysics Karolinska Institutet Stockholm Sweden; ^4^ Department of Clinical Biochemistry North West London Pathology Imperial College Healthcare NHS Trust Charing Cross Hospital London UK; ^5^ Department of Chemical Engineering University College London London UK; ^6^ West London Gynaecological Cancer Centre Hammersmith Hospital Imperial College NHS Trust London UK; ^7^ Department of Metabolism Digestion and Reproduction Imperial College London London UK

**Keywords:** design of experiments, lateral flow immunoassays, limits of detection, nanozymes

## Abstract

With the rise of non‐communicable diseases, lateral flow immunoassays (LFIAs) are well‐positioned to address the demand for disease monitoring. We present the use of peroxidase‐mimicking platinum nanozyme conjugates targeting three ovarian germ cell tumor markers (alpha‐fetoprotein (AFP), human chorionic gonadotropin (HCG), and cancer antigen 125 (CA125)) in LFIA. A “design of experiments” (DoE) approach was used to optimize antibody–nanozyme conjugation, superseding conventional “one‐factor‐at‐a‐time” optimization strategies, which neglect factor‐to‐factor interactions obscuring identification of optimal conditions. Crucial to disease monitoring, assays must demonstrate limits of detection (LoD) within clinically defined ranges and produce quantitative readouts. We address limitations of typical LoD calculations, which assume homoscedastic variance across marker concentrations, presenting an alternative model and applying it to assess LFIA performance, subsequently demonstrating clinically relevant LoDs in human serum. To robustly derive marker concentrations from LFIA readouts, the model was coupled with the resolution molecular concentration and, using patient samples, validated against laboratory gold standard values. AFP and HCG assays align well with gold standard measurements, with sensitivities of 87.5% and 100%, and specificities of 98.3% and 100%, respectively. This work outlines a development pipeline of a patient sample validated semiquantitative LFIA, utilizing DoE for streamlined optimization and improving modeling approaches to quantify performance in a manner representative of assay function.

## Graphical abstract

This study outlines the developmental pipeline of a multiplexed nanozyme‐based lateral flow immunoassay for the purpose of ovarian germ cell tumor detection. It demonstrates the application of a design of experiments optimization approach for nanozyme probe conjugate development. Furthermore, it implements a new modeling approach to calculate assay limit of detection and characterizes assay quantification when testing patient serum samples.

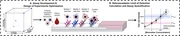

## 1 Introduction

With the global burden of disease shifting notably towards a relative increase in non‐communicable diseases (NCDs), there is a critical need for effective detection and surveillance of a plethora of conditions to enable life‐saving interventions [1, 2, 3, 4]. Cancers in particular, the second largest cause of NCD deaths worldwide, can potentially be prevented or more effectively managed with efficient diagnosis and monitoring [3, 4, 5, 6, 7]. Ovarian cancer (OC) is the sixth most common cancer in women in the UK, encompassing many subtypes, and is often linked to a poor prognosis [8, 9, 10, 11, 12]. Ovarian germ cell tumors (OGCTs) constitute one such subtype, that is nonepithelial and often affects younger women with fertility potential [13]. Close monitoring of OGCT response throughout and after treatment is critical to ensure that early action is taken in the case of continued tumor growth or recurrence, preventing adverse effects on patients’ health and quality of life [9, 14]. Current gold standard monitoring methods for OCs, alongside clinical examination and various imaging modalities (ultrasound, magnetic resonance imaging, computed tomography, etc.), currently rely on blood marker analysis using benchtop laboratory equipment [15, 16, 17]. This equipment is not only labor‐ and time‐intensive to operate, but also costly, consequently burdening healthcare systems globally, particularly those in low‐ to middle‐income countries [18, 19, 20].

**FIGURE 1:**
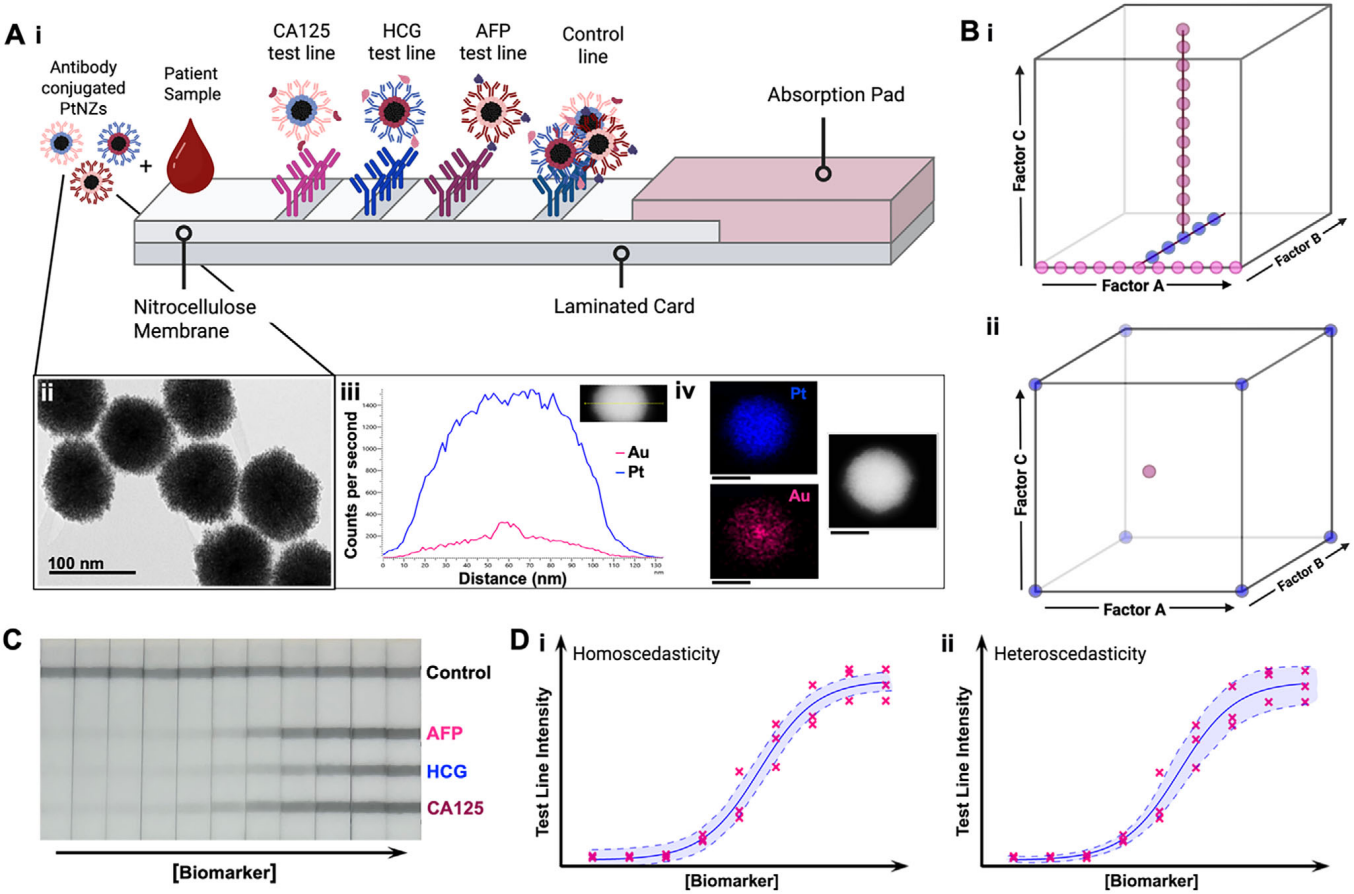
A. (i) Schematic of proposed multiplexed half‐dipstick lateral flow immunoassay (LFIA). (ii) TEM image and (iii) energy dispersive X‐ray (EDX) line map across the diameter of a single platinum nanozyme (PtNZ). (iv) TEM‐EDX spectroscopy map showing distribution of Pt and Au of a single PtNZ. The PtNZs were conjugated to antibodies to form the probe for the half‐dipstick immunoassay. 50 nm scale bar. B. Schematic outlining the difference between optimization approaches: (i) one factor at a time (OFAT) and (ii) design of experiments (DoE). OFAT approaches vary one experimental variable at a time, often requiring a greater number of experiments, in contrast to DoE approaches, which typically sample fewer experimental points across the design space allowing for the identification of factor‐to‐factor interactions in fewer experiments. C. Image of multiplexed half‐dipstick assays after being tested with human serum and serially diluted biomarker (AFP, HCG, and CA125) to generate a calibration curve standard. D. Calibration curve schematic comparing (i) current modeling methods used to calculate the limit of detection (LoD) with (ii) the method proposed herein, which accounts for heteroscedastic variance across different marker concentrations. All schematics are created using BioRender.

Paper‐based point‐of‐care (PoC) tools such as lateral flow immunoassays (LFIAs, Figure 1A) have emerged as a rapid and cost‐effective alternative for cancer biomarker detection [21, 22, 23]. However, their sensitivity has traditionally been limited, particularly when compared to gold standard methods (e.g., enzyme‐linked immunosorbent assay for protein or polymerase chain reaction for nucleic acid detection) [18, 24]. The drive for improved PoC sensitivity has resulted in the burgeoning development and application of various fields: from the class of affinity agents used, to the novel chemistries and methods employed to selectively modify and conjugate these bioreceptors, to innovative probes and reporter molecules [25, 26, 27]. However, some of these modifications can lead to increased costs either due to the requirement for complex external readers or due to the multistep probe manufacturing and conjugation approaches needed to functionalize the probe with affinity agents, consequently presenting a potential barrier to widespread adoption [28, 29].

It is therefore incumbent on developers to simplify LFIA manufacturing as much as possible, emphasizing careful and thorough optimization of key aspects of LFIA processes (such as probe optimization) to achieve device functionality in line with the desired clinical criterion. Typical optimization approaches rely on “one factor at a time” (OFAT) methods, which are often time‐ and resource‐intensive (due to varying only one factor in sequence) and do not assess the impact of interactions between the variables being optimized, thus limiting the discovery of the “true” optimum (Figure 1B). In contrast, “design of experiments” (DoE) approaches are significantly more efficient and informative, providing the ability to explore the influence of multiple factors simultaneously, including any interactions between them and their effect on a given response or output parameter [30, 31, 32]. This culminates in the identification of conditions that are likely to be closest to the “true” optimum and thus produce a better performing system. Although this method of optimization presents many advantages, there are very few examples of application in PoC device development [33, 34, 35].

The implementation of statistical methods is not only advantageous for guiding experimental design, but it is a critical means of evaluating assay functionality, specifically regarding its quantitative capability in the context of biomarker concentration monitoring. Calculating assay limits of detection (LoD) has long been held as a significant metric of analytical assay performance, with assay optimization generally conducted with the goal of minimizing the LoD below clinical requirements [36]. The LoD is typically defined as the lowest analyte concentration that can be reliably detected with a specified degree of confidence [37]. However, the method by which the LoD is calculated is often inconsistent, especially when compared to the first mathematical definition as put forward by Currie et al. [37, 38, 39, 40]. This results in a widely variable metric, which often does not consider the variability in signal intensities produced from testing samples covering a large analyte concentration range. Although previous works have intended to resolve this, as notably proposed by Holstein et al. who calculate a pooled standard deviation of the signal intensity of test samples, the approach taken, as the authors themselves reported, assumes that the variability in signal intensity is constant across all marker concentrations (Figure 1C,D) [41]. This negates any heteroscedasticity in its modeling, that is, that variability (or standard error) in test line signal intensities can change across analyte concentrations.

In addition to calculating the LoD as a measure of analytical sensitivity, the end application proposed for this work—monitoring OGCT marker levels—requires a metric that describes the semiquantitative capability of the assay. Biomarker concentration, especially when taken relatively to a patient's baseline, can be an indicator of disease status and responsiveness to treatment. Paper‐based colorimetric assays can be designed so that the intensity generated at the test line correlate to analyte concentration, but rarely are their semiquantitative capabilities commented on in a statistically consistent manner. The resolution molecular concentration (RMC), proposed by Wilson et al., was developed to identify whether assay signal intensities produced by samples at different marker concentrations are statistically significant [42]. This enables the identification of the smallest fold‐change in concentration between two samples, giving LFIA developers an idea of the resolution of their assay, that is, how well it is able to distinguish between samples with a small difference in marker concentration. Despite the importance of this metric for many LFIA applications beyond OGCTs, it has been rarely adopted in the PoC literature [43, 44].

Herein, we present a tri‐plexed LFIA for the detection of three OGCT biomarkers (human chorionic gonadotropin (HCG), alpha‐fetoprotein (AFP), and cancer antigen 125 (CA125)) in human serum (Figure 1Ai). The LIFA is developed using a DoE‐led approach to optimize the facile, one‐pot electrostatic conjugation of antibodies to peroxidase‐mimicking gold‐core platinum‐shell nanozymes (PtNZs). We propose an alternative method to model assay performance, which is used to derive an LoD value that is true to its initial definition, whilst considering the heteroscedasticity of signal intensity across the tested marker concentrations. The semiquantitative nature of the device is also evaluated using the RMC metric. The performance of the optimized multiplexed device was subsequently validated with patient and control serum samples and compared with gold standard measurement values.

## 2 Results and Discussions

### 2.1 Assays Incorporating Design of Experiments Optimized PtNZ–Antibody Conjugates Reach Clinically Relevant LoDs

A high‐throughput screen of several antibody pairs was conducted via in vitro plate‐based nanozyme‐linked immunosorbent assays (NLISAs, Figure S1). Serum‐stable peroxidase‐mimicking platinum core–shell nanozymes were employed as probes in place of the commonly used horseradish peroxidase enzyme. The PtNZs as described by Loynachan et al. consist of a ≈15 nm gold core over which a platinum shell is grown through the reduction of a platinum precursor [45]. These particles were visualized by TEM‐EDX and were consistent with the previously reported porous urchin‐like structure (Figure 1Aii–iv). Substituting the typical biological peroxidase probes with PtNZs allows for superior assay sensitivity due to greater catalytic efficiency with a *k*_cat_ four orders of magnitude higher than HRP (Figure S2). This is additional to increased stability and batch‐to‐batch reproducibility reported in the literature [45, 46].

The NLISAs demonstrated that the antibodies were capable of reaching clinically relevant LoD cut‐offs (AFP < 14 ng/mL, HCG < 5–10 mIU/mL, CA125 < 35 IU/mL, Figure S1I–L), though this had yet to be translated to the paper‐based format [47, 48, 49, 50, 51, 52]. Although plate‐based assays are useful for screening antibody pairs due to their high‐throughput capability, when considering our end application, the long incubation times, lack of capillary flow action and polystyrene material do not resemble the conditions typically expected for LFIAs thus making it an unsuitable medium for assay optimization. We therefore opted to optimize the assay in the typical nitrocellulose membrane‐based half‐dipstick assay format. Although there are many key factors influencing the performance of LFIAs such as membrane material and flow rate, the conjugation of the affinity agent to the probe is often the most foundational component of LFIA function. This is due to its influence on marker detection in a manner which is largely independent from strip architecture and therefore formed the natural starting point for optimization in this work [53, 54]. Ensuring effective conjugation maximizes signal generation at the test line due to improved capture of the marker of interest. It also minimizes nonspecific binding (NSB) between the conjugate and membrane or interfering sample components [53, 54]. This culminates in the generation of more intense test line signals solely in the presence of the target marker, limiting false positive results and improving analytical sensitivity [55]. We opted for an electrostatic conjugation approach due to its simple, robust, one‐pot nature [45]. However, this requires careful optimization due to the many parameters influencing the success of attachment and orientation of antibodies on the NP surface.

In a typical conjugation of PtNZs, detection antibodies are added to the nanoparticles in a conjugate buffer (at a specified pH and molarity) and incubated under agitation at room temperature, followed by the addition of a blocking agent. The PtNZ conjugates are subsequently washed to remove excess reagents and are then characterized by dynamic light scattering (DLS), where a slight increase in size is expected (Figure S3A) [45]. Five main factors that have been shown to widely affect the electrostatic conjugation process and the resulting conjugate performance in LFIA signal generation (Signal and NSB) are outlined in Table 1 [56, 57, 58, 59, 60, 61].

**TABLE 1:** List of five factors influencing the electrostatic conjugation of platinum nanozyme (PtNZ) probes to antibodies. The column on the right lists the ranges of each factor explored in the design of experiments (DoE) optimization process. Values in the brackets represent the center point.

Factors influencing conjugation	Range of factors		
pH of conjugation buffer	• Anti‐AFP: 5–7.5 (6.25) • Anti‐HCG: 4.5–7.5 (6) • Anti‐CA125: 5.8–8.2 (7)		
Ionic strength of buffer	20–250 mm (135 mm)		
Equivalence of Antibodies per PtNZ	100–1000 (550)		
Blocking agent	BSA	PVP	β‐Casein
Concentration of blocking agent	0.2%–2% (1.1%)		

One of the key factors influencing the conjugation is pH. pH plays an essential role in manipulating the charge distribution across antibody surfaces and has been reported to influence the surface charge of PtNZs [56, 62, 63]. This not only determines the ability of an antibody to electrostatically adhere to a substrate, but also influences its orientation, thus affecting the availability and density of immunoglobulin binding regions (Fab) on the probe surface [56]. To add further complexity to the optimization process, the impact of pH on an antibody's overall charge is heavily reliant on its amino acid composition, as signified by antibodies’ varying isoelectric points, i.e., the pH at which an immunoglobulin has no net charge [64]. The pH of the conjugate buffer must therefore be optimized individually for each antibody. For our use‐case, optimizing the pH of the conjugate mixture to the point where antibodies added have a slightly net positive charge would complementarily adsorb to the negatively charged PVP‐capped PtNZs [56, 65, 66]. However, this must be fine‐tuned as increasing the number of positively charged pockets on the antibody surface may induce antibody bridging across multiple PtNZs, promoting aggregation or resulting in permanent antibody conformational change [56, 67, 68, 69]. This can also increase the likelihood of NSB to nontarget species in serum or result in a weaker test line as a consequence of the increasingly randomized antibody orientation on the probe surface [56, 70]. For each antibody conjugation, acidic pH buffers (ranging from 4.5 to 6) were screened to determine the starting point of the pH range incorporated into the DoE, with the lowest pH limit defined as that leading to the onset of dramatic particle aggregation, assessed through visual observation (Table 1; Figure S3B). Particles conjugated at higher pH were characterized by DLS to ensure monodispersity, with a PDI < 0.15 (Figure S3C–E). Alongside pH, we considered the ionic strength of the conjugation buffer as a key factor to optimize. Ionic strength is critical in manipulating the Debye length, which defines the thickness of the repulsive electrical double layer (EDL) from the surface of the nanoparticle. Buffers of high ionic strength often result in a smaller EDL, which can lead to compromised particle colloidal stability and induce aggregation [59, 71, 72]. However, this parameter can also influence antibody charge and stability, subsequently impacting the rate of immunoglobulin adsorption onto the probe surface and emphasizing the need for careful optimization [59].

The third factor requiring optimization is the antibody equivalence, i.e., the number of antibodies added per PtNZ. Theoretically, greater antibody densities on the conjugate surface increase the number of binding sites and subsequent binding events, potentially leading to greater test line intensities and low NSB due to adequate coverage of bare particle surfaces [73]. However, too high a concentration (other than being an unnecessary excess) can also facilitate PtNZ aggregation or NSB due to antibody complexes forming with nontarget analyte components in the test matrix [57, 58]. Antibody aggregation can also occur in high concentrations, potentially stripping antibodies away from the probe surface, resulting in a reduction in assay signal. Conversely, PtNZ surfaces that are sparsely coated with antibodies would lead to the reduction of binding events and increase the area of bare PtNZ surfaces that can contribute to NSB [55, 57, 73, 74]. A potential method of mitigating this is through the incorporation of blocking agents, paying close attention to the concentrations used in the solution, thus forming the fourth and fifth variables of interest incorporated in the DoE. Nanoparticle blocking is an essential part of the conjugation process as it provides coverage of bare, unoccupied PtNZ surfaces, which can otherwise potentially form electrostatic interactions with the test line or random sample matrix components, leading to the generation of false positive signals [60, 75]. This can also elevate the background signal in catalytically amplified assays, thus reducing the relative test line intensity. Three different blocking agents, most utilized in the literature for PtNZ probes conjugation, and encompassing different sizes and isoelectric points, were therefore screened: bovine serum albumin (BSA), β‐casein, and polyvinylpyrrolidone (PVP, 10 kDa) [45, 76, 77, 78, 79, 80].

The concentration of these blocking agents was also included in the DoE model to ascertain the amount needed to ensure adequate coverage of bare PtNZ surfaces whilst also not being in great excess to the point of potentially displacing antibodies, inducing aggregation, or causing steric hindrance, consequently inhibiting binding events.

The incorporation of the blocking agent also enabled us to determine the range of antibody equivalences (per PtNZ) to be optimized via the DoE, as fewer antibodies could be used, thus demonstrating the necessity of optimizing these parameters simultaneously. Given that the PtNZs incorporated herein have a diameter of ≈120 nm, an estimate of 550 antibodies per PtNZ would be required to form a theoretical monolayer (Appendix S1). The antibody equivalence range incorporated into the DoE was therefore 100–1000 antibodies per PtNZ, with 550 antibodies taken as a midpoint, with the assumption that not all antibodies added to the conjugate mixture would successfully adsorb onto the probe.

As suggested earlier, and when considering the conjugation factors mentioned, it is highly likely that these variables not only influence the electrostatic conjugation process in isolation, but also influence overall conjugate performance in the assay through reciprocal interaction. High antibody equivalences may not be necessary, for example, when using acidic conjugation buffers, where antibodies are likely to be more positively charged, in comparison to buffers at a higher pH. Such interactions between parameters must be fully considered and optimized to achieve a highly efficient probe conjugate. This can be easily incorporated with a DoE approach in contrast to the often‐employed, time‐consuming OFAT optimization method.

The factors that we selected as essential for the optimization of the electrostatic functionalization of PtNZs with detection antibodies encompass four continuous and one categorical variable (Table 1). Critically, the DoE model must consider quadratic interactions (i.e., nonlinear trends for continuous variables) and two‐way interactions between parameters (Figure S3F). Traditional DoE designs such as factorial, central composite, and box Behnken designs, which typically consider such interactions cannot feasibly accommodate the three‐level categorical variable included herein (the blocking agent) [30, 81]. We therefore utilized an I‐optimal experimental design implemented in the statistical design software *JMP* to generate a custom design platform to accommodate all the requirements whilst minimizing the number of experiments (runs) [82, 83, 84]. Optimality criteria are mathematical functions that enable the identification of the best experiments to run in a manner that provides maximal information for a given target. In an I‐optimality criterion, experiments are chosen with the aim of minimizing average predictive variance; that is, so that predictions that the model makes are as accurate as possible over the range of variables being studied [85, 86]. The continuous factors included in the DoE consisted of two levels (a high and a low value, e.g., pH 4.5 and pH 7.5) as well as a center point (e.g., pH 6) to map out quadratic effects and estimate curvature.

Using this criterion in a custom design generated 28 combinations of conjugation conditions, inclusive of some repeat conjugation conditions at the center point to account for experimental variability (Figure S4A–C) [84]. All possible interactions between any two of the optimization factors (also known as second‐order interactions) were considered in the design. The outputs (also known as the responses) for which the conjugate is being optimized were determined to be the signal at the LFIA test line in the presence of the target analyte and the NSB in its absence.

All conjugates were prepared as per the custom design recommendations over a period of two days. The resulting probes were then checked for size and polydispersity via DLS, which were shown to be monodispersed (polydispersity index < 0.15, Figure S3C–E). All conjugated PtNZs were then tested in half‐dipstick LFIAs in spiked and unspiked pooled human serum at a fixed concentration (100 pm) to determine the signal and NSB generated at the test line. Images of the strips were taken and analyzed using image analysis software to attribute quantitative values to observed test line intensities. A half‐dipstick assay differs from a full LFIA in that it does not include the sample or conjugate pads, rather the solution containing sample and conjugates is added directly to the nitrocellulose membrane. It is commonly employed in the earlier developmental and optimization stages of LFIAs [45, 60, 78]. This simplified form enables the facile testing of different conjugates, isolating the optimization of the PtNZs conjugates from the rest of the LFIA components, thus mitigating the need to assess conjugate pad release kinetics until an optimized probe conjugate is found. Furthermore, the use of serum means that there is little need for sample pads at this stage, which filters interferants typically expected from whole blood samples such as erythrocytes.

**FIGURE 2:**
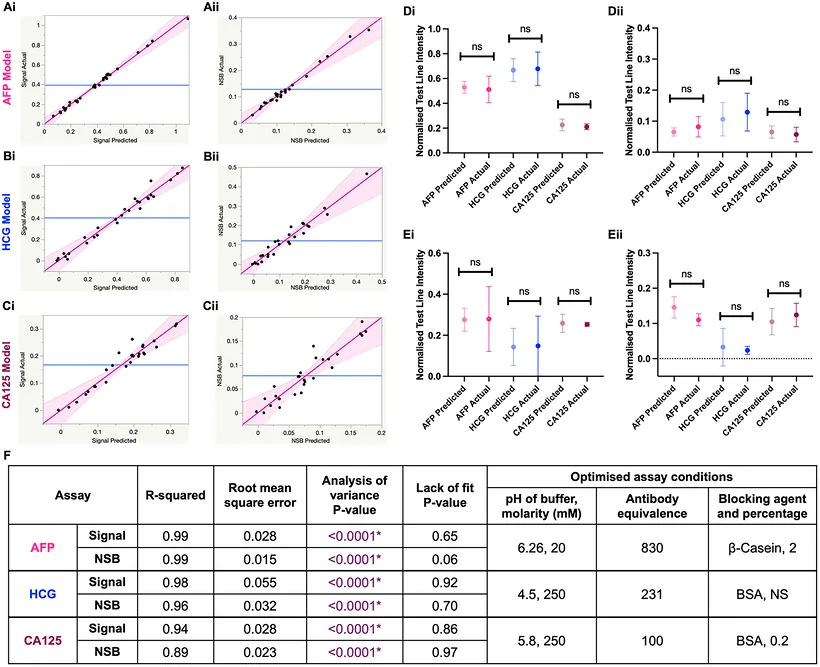
Actual (*Y*‐axis) experimental values of (i) signal and (ii) nonspecific binding (NSB) resulting from testing platinum nanozyme (PtNZ)–antibody conjugates obtained from different conjugation conditions in half‐dipstick immunoassays plotted against model‐predicted (*X*‐axis) values for the A. AFP, B. HCG, and C. CA125 assays (*N* = 1, *n* = 3). Experimental results of D. model optimized, and E. high variance conjugations compared to model‐predicted values for (i) signal and (ii) NSB. Paired *t*‐tests were used to test for significant difference between actual and predicted values. ns‐ nonsignificant difference. This refers specifically to the percentage of the BSA blocking agent not being a significant contributor to the HCG model and 0.2 wt%/vol% of BSA was used in the optimal conjugate. (Data shown as mean ± S.D., D. *N* = 3, *n* = 3, E. *N* = 1, *n* = 3, where *N* indicates the number of conjugation batches, i.e., experimental repeats, and *n* is the number of half‐dipstick strips these conjugates were tested on, i.e., technical repeats.) F. Table outlining the parameters of the signal and nonspecific binding (NSB) models fitted for each of the three assays (AFP, HCG, and CA125), as well as the model‐derived optimized conjugation conditions for each assay.

The signal and NSB data obtained for each set of conjugations were then plotted and modeled using least squares regression, generating a response surface method (RSM) model. Each conjugation parameter included in the RSM model was tested for significance, and those deemed insignificant (*p* > 0.05) were removed from the model (Figure S4D–I). The refined model‐predicted response values for signal and NSB were then plotted against the actual experimental values, demonstrating concordance between the experimental data and model‐generated predictions as evidenced by the R‐squared value and relatively low RMSE (Figure 2A–C,F). The models for each assay were subsequently assessed using a one‐way analysis of variance (ANOVA) to determine whether the proportion of variation in the experimental data is significantly explained by the adjusted model. This confirmed that the models generated for all three conjugates (AFP, HCG, and CA125) were able to significantly (*p*‐value < 0.05) explain the variation in the responses (signal and NSB). Furthermore, the models were shown to have an insignificant lack of fit, whereby the model error is shown to be insignificant (*p* > 0.05) when compared to the pure error variation resulting from the replicated conjugations.

Having established a good‐fitting model for the experimental data, further validation was conducted. First, optimal conjugation conditions were derived from the model by setting the desirability criteria so that signal at the test line is maximized while NSB is minimized (Figure S5). PtNZs were subsequently conjugated under model‐recommended conditions and tested in half dipstick assays to assess if the signal and NSB values generated match model predictions. In all cases, signal and NSB at test lines appeared to fall within the model‐predicted ranges (Figure 2D). To determine the scope of the predictive capability of the model, further experiments were conducted at regions where the predictive model uncertainty is greater (as determined by JMP). This again demonstrated that model‐predicted values closely match experimental values (Figure 2E).

Having validated the models for the three assay conjugates, we can more confidently analyze trends in the data. A closer examination of the models reveals that varying emphasis is placed on different parameters, and the weight of parameters in the models strongly depends on the different antibodies used during conjugation (Figure S4D–F). This highlights the extensive variation in the conditions needed for PtNZs functionalization between different antibodies within the same antibody class (mouse‐derived IgG1), thereby emphasizing the importance of efficient and thorough optimization for each antibody. For all conjugations, primary factors such as pH, antibody equivalence, and blocking agent were significantly influential in signal and NSB generation, with ionic strength and the percentage of blocking agent varying in significance from assay to assay. The percentage of blocking agent was found not to be significant in the HCG model (Figure 2F), so we resorted to using the lowest concentration of 0.2% w/v. All three models also highlight the significant contribution of second‐order interactions, reaffirming the importance of accounting for these factors in optimization approaches, emphasizing the limitations of conventional OFAT methods (Figure S4D–I). Some of the important interactions observed in the HCG model included the one between buffer pH and antibody equivalence. The effect that this interaction had on the output parameters was such that under more acidic conditions stronger signal and NSB intensities were observed and appeared to increase further at greater antibody equivalences. However, under more alkaline conditions there appeared to be little change in signal intensity and NSB regardless of antibody equivalence (Figure S6A). This can be explained by the fact that if the conjugation buffer pH is not below the antibody isoelectric point, antibodies would not be appropriately charged to electrostatically adsorb onto the PtNZ surface, regardless of how many antibodies were introduced.

In the AFP and CA125 models, the interaction between blocking agent type and buffer pH was noted, amongst others. The incorporation of PVP as a blocking agent appeared to make the conjugation system more sensitive to pH, as the range in signal and NSB increased significantly when going from acidic to more alkaline pH. Conversely, the addition of ß‐casein seemed to decrease the effect of pH on signal and NSB (Figure S6B,C). A potential reason for this finding may be due to the isoelectric point of the blocking agents. PVP, an organic nonionic polymer, is unaffected by pH directly, especially when compared to ß‐casein (pH 4.6–5.1) and BSA (pH 4.7–5.3) protein blocking [87]. It is therefore likely that there is little interaction occurring between PVP and the exposed NP surfaces, and that interaction is limited to neighboring antibodies. Thus, the influence of pH observed on signal and NSB in PVP conjugates can perhaps be attributed to the direct impact of pH on antibody adsorption to the PtNZ surface. Conversely, the reportedly higher isoelectric point of ß‐casein and BSA increases the likelihood of adsorption to the negatively charged bare particle surface, resulting in both reduced signal and NSB intensities. This could also explain the significance of the interaction between blocking agent and antibody equivalence (Figure S6D). In the presence of ß‐casein blocking, signal intensity appeared to increase with the addition of greater antibody concentrations. Little difference in NSB was noted despite that. However, with the addition of PVP, both signal and NSB increased proportionally, suggesting that PVP is an ineffective blocking agent.

### 2.2 Heteroscedastic Variance Incorporated Into Limit of Detection Calculations

**FIGURE 3:**
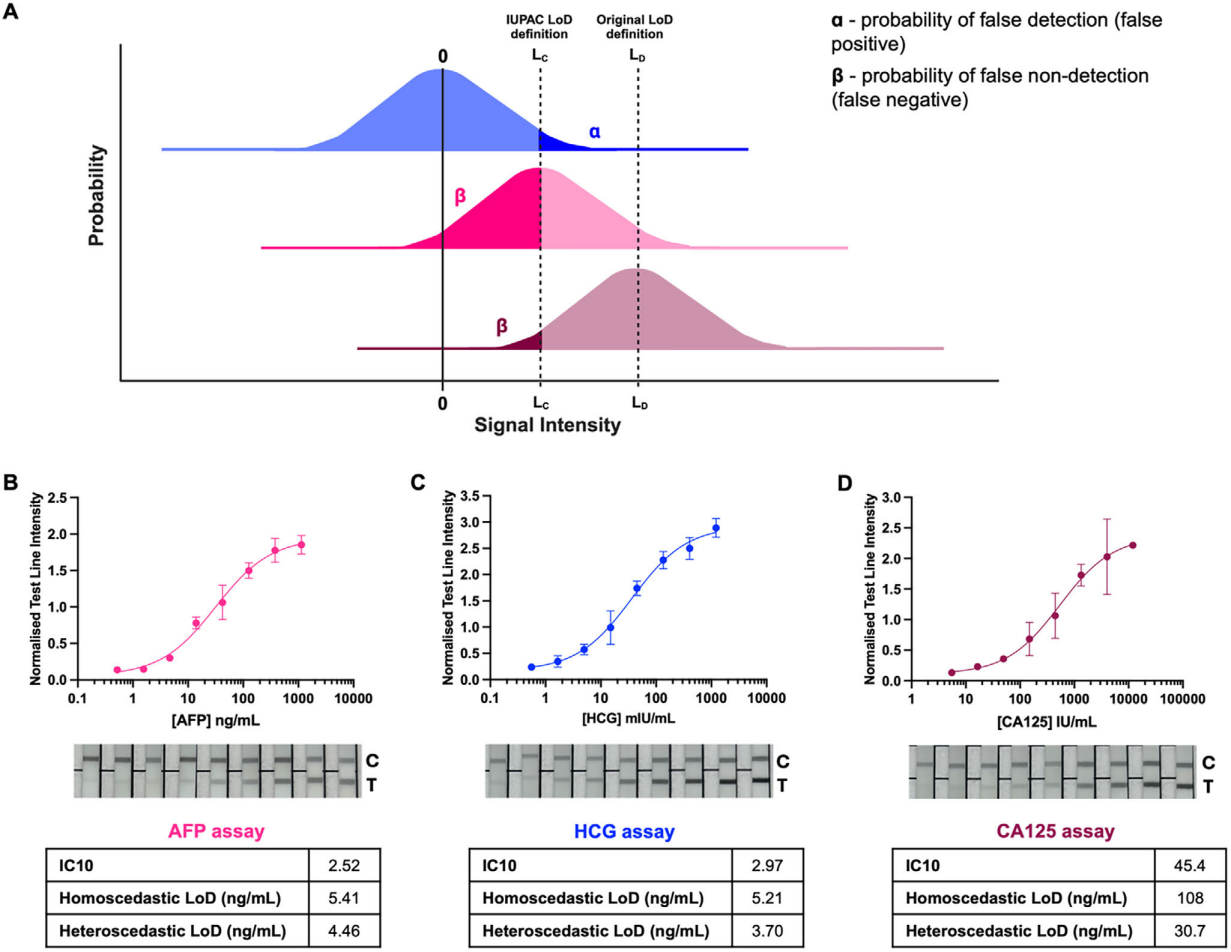
A. Schematic outlining the method by which the detection limit (LD) is assigned. In the original definition, the signal distribution of blank samples is plotted (blue), and an α limit (probability of false positive) is assigned. The corresponding signal intensity is known as the critical limit (*L*_c_). The *L*_c_ is then used to fix β, which is the probability of false nondetection, from which a distribution can be plotted (maroon), and the detection limit signal, L_D_ derived. This method is contrasted to the IUPAC definition, where the limit of detection (LoD) definition is equivalent to that of the *L*_c_, causing the β value or false negative rate to be much higher, when considering its signal distribution (pink). Calibration curves relative to the assays for B. AFP, C. HCG, and D. CA125, where serial dilutions of the protein marker are spiked in serum and mixed with the respective DoE‐optimized antibody–platinum nanozyme (PtNZ) conjugate. (Inset) Images of corresponding half‐dipstick strips are shown. The numerical assay performance is outlined in the table comparing the different modalities (homoscedastic and heteroscedastic) of determining the limit of detection (LoD). C ‐ control line, T ‐ test line. Data shown as mean ± S.D., *N* = 1 *n* = 3, where *N* indicates the number of independent experiments and *n* is the number of technical replicates (half‐dipstick strips) at each marker concentration. Schematic is created using BioRender.

To determine each assay's LoD as a marker of analytical sensitivity, the model‐recommended optimal conjugates and their respective half‐dipstick assays were tested with the appropriate marker, serially diluted in pooled human serum. The LoD is defined following Currie's seminal framework [37]. Initially, the critical limit (*L*_C_​) is determined in the signal domain (i.e., test line intensity), representing a threshold that ensures a probability α of false detection (i.e., false positive) (Figure 3A). Based on *L*_C_, the detection limit (*L*_D_) is derived in the signal domain, ensuring a probability β of false nondetection (i.e., false negative). This *L*_D_ is subsequently translated from the signal domain into the concentration domain (i.e., concentration of the antigen), utilizing a regression model, to establish the LoD. This definition of LoD is also used by Holstein et al. [41]

Unfortunately, this definition of LoD is not applied consistently, as highlighted by Faber [39]. For example, the IUPAC definition of LoD corresponds to the above definition of *L*_C_ [38]. While the IUPAC approach inherently yields a lower LoD value compared to the original definition, its primary limitation is relying solely on the distribution of blank measurements, making it unsuitable for benchmarking diagnostic tests. In contrast, the original definition of LoD incorporates the distribution of measurements for both blank samples and samples containing the analyte, providing a more comprehensive approach to evaluating detection performance (Figure 3A) [39].

In this study, we will set both the α‐level and β‐level to 0.05, meaning that we accept a 5% probability of false detection of blank samples and a 5% probability of false nondetection of samples containing an analyte concentration corresponding to the LoD. For the regression model relating test line intensities to analyte concentrations, we employ a gaussian processes (GP) regression model [88]. This approach differs from the four‐ or five‐parameter sigmoidal models described by Holstein et al. that are frequently applied when modeling LFIA data [41, 89, 90, 91].

The GP regression model is a nonparametric, probabilistic approach that models the relationship between test line intensities and analyte concentrations. Instead of fitting a fixed functional form, the GP defines a distribution over possible functions, characterized by a mean function and a covariance (kernel) function [88]. The kernel function encodes assumptions about the smoothness and structure of the relationship, allowing the GP to capture complex, nonlinear patterns in the data. Given training data, the GP provides predictions for new inputs along with uncertainty estimates, making it particularly well‐suited for modeling measurement data where uncertainty plays a critical role, subsequently accounting for heteroscedasticity in the data that is otherwise omitted (see Appendixes S2 and S3).

For LoD calculation, we combined the heteroscedastic GP fit with a resampling engine that simulates full calibration datasets per concentration level (parametric/residual resampling) and refits the GP for each draw. In each bootstrap, the blank distribution yields a critical limit *L*_C_ (one‐sided *t* at α  =  0.05) with a numerical standard deviation floor and shrinkage of per‐level standard deviations toward a pooled low‐end standard deviation to avoid degenerate small‐*n* levels. The detection probability *P*(*Y* > *L*_C_∣*x*) from the GP predictive distribution is then evaluated over *x*; the LoD is the smallest *x* where *P* ≥ 0.95. We report mean LoD across *B*  =  1000 bootstraps; *L_C_* is summarized analogously. This procedure preserves Currie's two‐error definition while accounting for heteroscedasticity and model uncertainty. Minor numerical shifts in LoD are expected between runs due to resampling.

Once the GP regression model described was fitted to the calibration curve assays incorporating the DoE‐optimized conjugates, we were able to determine that for both the AFP and HCG assays, the LoD values were below the clinical cutoffs of 20 ng/mL and 5–10 mIU/mL, respectively, indicating that these assays were ready for multiplexing (Figure 3B,C). In the case of CA125, unlike the heteroscedastic LoD calculation, the homoscedastic LoD did not appear to meet the clinical requirement of 35 IU/mL due to the averaging of variance in the model fitted, masking the true sensitivity of the device and heterogeneity of signal generation across different marker concentrations (Figure 3D). The IC10—the marker concentration corresponding to 10% of the maximum signal—was also calculated from the calibration curve as is traditionally reported in literature [90, 92].

Nevertheless, with the DoE optimized conjugates’ promising performance in the LFIA format, multiplexing the three assays and fine‐tuning their performance formed the next critical step. Other crucial components of a LFIA that require optimization can be classified into two broad categories: components that maximize test line signal intensity, and those that reduce background noise in the surrounding membrane. Increasing the incidence of antigen binding events by increasing capture antibodies and detection probes can, for example, intensify the test line signal. However, careful optimization of the probe concentration was required to minimize excess NPs settling in the membrane resulting in an elevated background signal, which can diminish the relative test line intensity. The use of sample diluents and wash buffers with protein, polymer and surfactant additives can also help mitigate this by pushing excess probes along the strip, subsequently preventing stagnation [93, 94, 95]. Furthermore, they can neutralize interfering sample components and regulate sample flow, thereby dictating the length of time during which sample components interact before reaching the test line and absorbance pad [93, 96]. In addition to the use of wash buffers, sample flow can also be controlled by varying test line height, using nitrocellulose membranes with different degrees of porosity, and changing the absorbent pad thickness [97, 98, 99].

### 2.3 Three Ovarian Germ Cell Tumor Assays Are Integrated Into a Single Multiplexed Half‐Dipstick Lateral Flow Device

**FIGURE 4:**
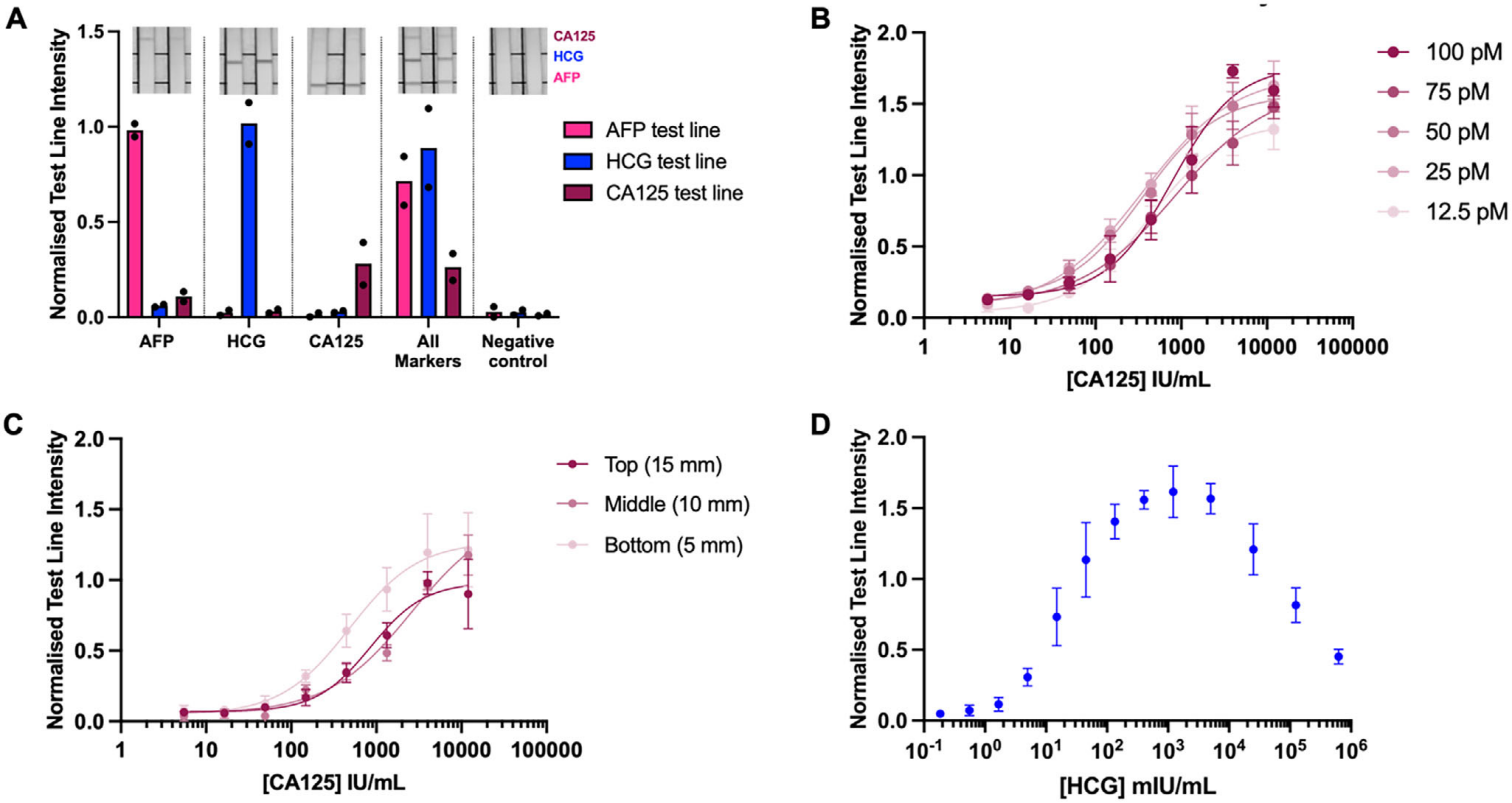
A. Multiplexed assay cross‐reactivity screen. Each marker was spiked individually in the presence of all conjugates and compared to a negative control and a positive control where all markers where present (*N* = 1, *n* = 2). B. Calibration curves plots of serum antigen concentration and corresponding assay test line intensities for CA125 half‐dipstick assay tested with three different conjugate concentrations (100 pm, 75 pm, 50 pm, 25 pm, and 12.5 pm). C. Calibration curves for CA125 dipstick assays with test lines printed at three different distances (15 mm, 10 mm, 5 mm from bottom of membrane). D. Calibration curve of HCG assay at very high HCG concentrations starting from 1,000,000 mIU/mL to elucidate the hook effect. Data shown in panels B,–D as mean ± S.D., *N* = 1 *n* = 3, where *N* indicates the number of independent experiments and *n* indicates the number of technical replicates (half‐dipstick strips) at each marker concentration.

In the initial iteration of the multiplexed half‐dipstick assay, we positioned each assay test line on the basis of the LoDs determined previously with the CA125 assay placed closest to the absorbent pad (15 mm from the bottom of the nitrocellulose membrane), followed by the HCG (10 mm), and AFP (5 mm) assays. This was based on the assumption that by placing the CA125 assay furthest along the membrane, more time is given for the sample and PtNZ conjugates to interact resulting in improved test line capture of antigen–conjugate complexes [99, 100]. We confirmed that there was no significant cross‐reactivity between the three assays, when all three PtNZ conjugates were added simultaneously in serum, but the LoDs were far from reaching the clinically desired values (Figure 4A; Figure S7) for the HCG and CA125 assays. Subsequently, we optimized the PtNZ concentration as the cumulative effect of combining the PtNZ conjugates from the single‐plexed assays led to a severe elevation of background signal. The total PtNZ concentration was therefore reduced from 300 pm (100 pm for each conjugate) to 95 pm (25 pm, 20 pm, and 50 pm for CA125, HCG, and AFP, respectively), which improved CA125 detection without significant impact on the HCG or AFP assays (Figure 4B; Figure S8B).

To further reduce the CA125 LoD, previous assumptions about assay performance in relation to test line height were challenged. We observed an improvement in assay performance when test lines were moved closer to the sample (Figure 4C; Figure S8A). Bartosh et al. demonstrated that the influence of test line height on assay performance is often a balance between assay kinetics and membrane flow [97]. Membrane capillary flow rate decreases as the sample moves up the nitrocellulose membrane and although placing the test line further away allows more time for conjugate–antigen interactions to occur, the same is true for nonspecific binding of the conjugate to the strip. Hence, in the case of an antibody with fast binding kinetics, increasing the test line height can eventually lead to worsening assay performance due to higher background signal generation with little improvement in the positive antigen specific signal [101]. The stagnating flow can also result in a reduction of PtNZ‐biomarker complexes reaching the test line, thus reducing signal intensity. As a result, the CA125 assay was placed at the bottom of the strip (5 mm), whilst decreasing the distance between the test lines shifting the HCG (8 mm) and AFP (11 mm) assays also closer to the sample source.

A similar rationale, based on improving sample flow and PtNZ clearance through the strip as well as minimizing sample interferents, led to the subsequent exploration of incorporating different additives and surfactant concentrations in the sample and PtNZ dilution buffers. Up to this point the diluent buffers consisted of phosphate buffered saline and Tween20 at a low concentration (0.05% v/v). Here, we found that the incorporation of higher Tween20 concentrations in the sample diluent (0.75 v/v%), along with ß‐casein as an additive in both the sample and PtNZs diluents, resulted in an improvement in signal intensity, across different target analyte concentrations (Figure S9A–E).

Given the clinical end application, the optimized assay was then used to assess the protein biomarker concentrations at which the hook effect is notable. The hook effect occurs at high protein concentrations, where capture and detection antibodies binding sites are saturated and can no longer form a complex immobilized at the test line. This forms a potential barrier to translation, particularly for semiquantitive PoC devices. The assay most prominently affected by this artefact in clinically relevant ranges is the HCG assay, where a decline in test line intensity is evident after 5000 mIU/mL (Figure 4D; Figure S8C). Conducting a single 100‐fold dilution can be employed to remove this interference and is widely used in the field [102, 103, 104, 105]. The hook effect was not noted in the CA125 assay, where dilutions were conducted starting from a concentration of 20,000 IU/mL and with the highest concentration reported for OGCTs in literature being 7778 IU/mL [106]. However, the effect was noted (to a much lesser extent than HCG) in the AFP assay between 270,000 and 810,000 ng/mL, where the highest reported concentration in the literature thus far for OGCTs is 4,233,000 ng/mL, suggesting that it may be of benefit to conduct serial dilutions of samples to mitigate AFP hook effects as well [107]. To identify whether a sample needs further dilution in practice, some studies have shown that the existence of the hook effect can be monitored by the speed and order of the formation of test and control lines [108, 109]. This can be monitored when using patient samples to guide the necessity of conducting a 100‐fold dilution using a supplied sample dilution kit, however, further studies will be required to optimize test operation. Since we envision these tests to be performed by trained healthcare professionals at the PoC, particularly due to the implications associated with the results, concerns regarding variable patient operation are minimized. Furthermore, dilution effect across the other unaffected markers can be mitigated by taking the value of the assay that yields a higher test line signal intensity. This ensures that the analytical sensitivity of all assays is not negatively affected by the 100‐fold dilution.

With the end application of this device being the PoC setting, peripheral fingerpick blood samples will likely be obtained using traditional lancets, which can withdraw ∼120 µL of whole blood [110], some of which will likely be lost during the filtration phase via sample pad. We therefore set out to explore the minimum sample volume required for the device to retain its functionality by screening different volumes of spiked serum with a fixed target antigen concentration. We observed that at least 24 µL of serum is required for optimal assay function (Figure S9F), which should, in future, give room for the filtration of whole blood components, as serum makesup to 40%–50% of whole blood volume [111]. To prevent interference from any of the whole blood components, such as erythrocytes, sample pretreatment filtration pads have been described in the literature, which separate whole blood components, preventing interferants from reaching the test line and influencing assay performance [112, 113, 114]. The total time taken to run the test is also key to successful device translation, with the World Health Organization's (WHO) REASSURED criteria for PoC tools suggesting that they should ideally be conducted in under an hour [115]. By screening two different assay incubation times we determined that 35 min is the total time required to conduct the assay without compromising test line signal intensity (Figure S10).

### 2.4 Patient Sample Validation and Statistical Analysis of Optimized Assay Indicate Clinical Relevance

**FIGURE 5:**
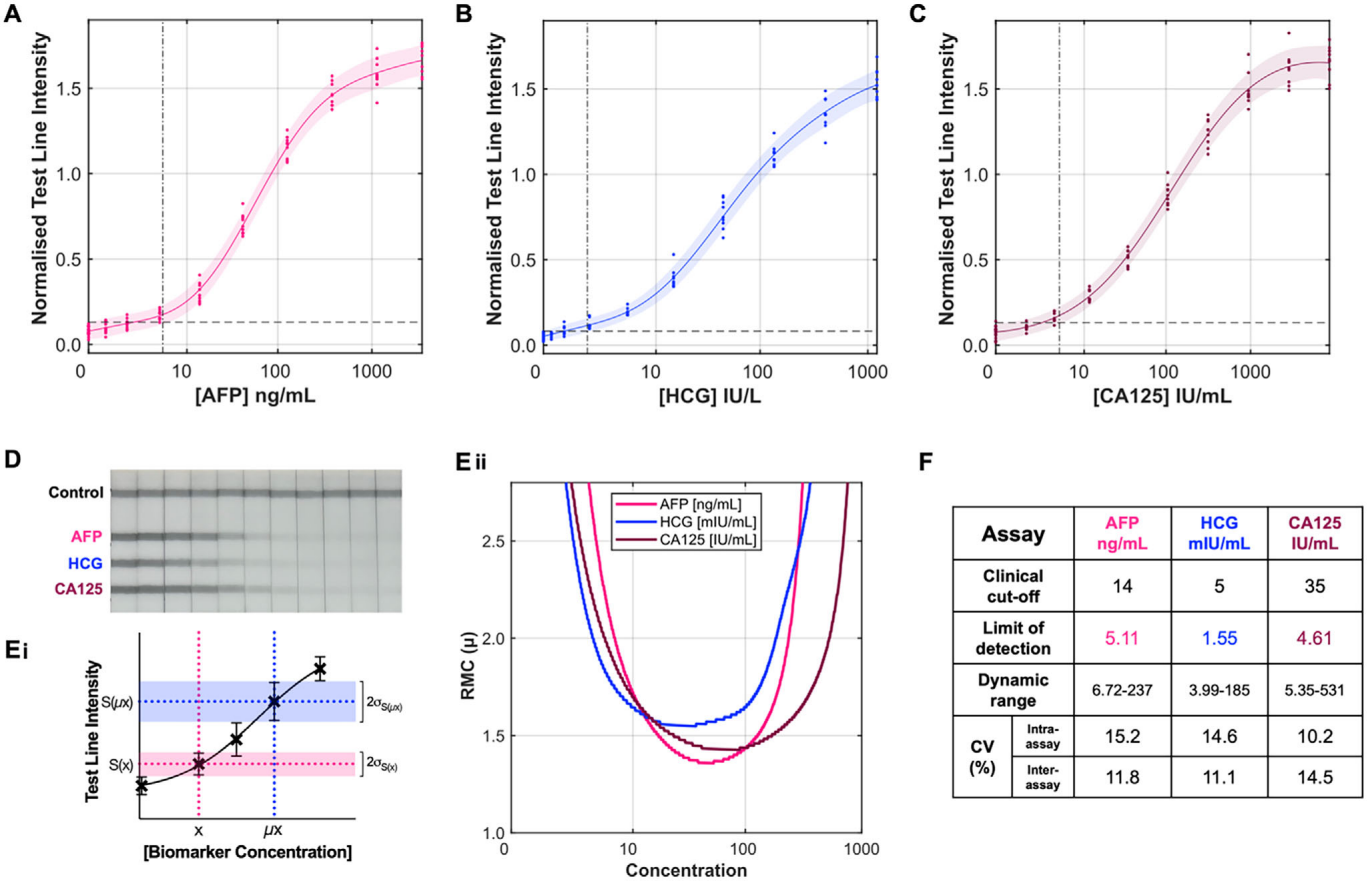
Multiplexed calibration curve assays of A. AFP assay using World Health Organization (WHO) standards, B. HCG assay using the sixth international WHO standard, and C. CA125 assay using a commercial CA125 standard. Graphs show individual data plots (*N* = 3, *n* = 3) fitted with the heteroscedastic model. *N* indicates the number of independent experiments conducted, including conjugation batches and half‐dipstick assay preparation; *n* refers to the number of half‐dipstick strips this was tested on at each marker concentration (i.e., technical replicates). Shaded regions represent 95% confidence interval. D. Image of the multiplexed calibration curve assay. E. (i) Schematic explaining the resolution of molecular concentration (RMC) whereby the signals obtained from an assay corresponding to two different concentrations (*x*) and (*µx*) are significantly different with 95% confidence. The RMC is then obtained by plotting *µ* as a function of (*x*) where at a given concentration, *µ*(*x*) is the smallest fold change in analyte concentration that is significantly distinguishable from (*x*), also at 95% confidence. (ii) Plot of RMC changes across different marker concentrations. F. Numerical calibration curve parameters corresponding to panels A–C of the optimized assays. The dynamic range refers to the marker concentrations corresponding to the region where the RMC ≤ 2. The limit of detection is calculated using the heteroscedastic method. Schematic in panel E(i) was created using Prism GraphPad.

The triplexed half‐dipstick LFIA was then tested in serially diluted AFP and HCG WHO standards, alongside commercial CA125 standards in pooled human serum to determine the LoD of the final optimized assay. All three assays were found to have LoDs below or within the clinical cut‐off range, as well as inter‐assay and intra‐assay coefficients of variation (CV) that were below ≤ 15.2%, indicating robustness and readiness for patient samples analysis (Figure 5A–C,F). Furthermore, given that one of the device aims is to quantify marker concentration, the RMC was derived to determine the smallest fold change in analyte concentration that can be statistically distinguished at a given concentration level. This was conducted following the method outlined by Wilson et al. using a 95% confidence interval, with the calibration curve model incorporating heteroscedasticity to account for variations in measurement noise across different concentrations [42]. This demonstrated that the smallest fold change in concentration varied between 1.36 and 1.55, which corresponded to the linear region of the calibration curves (Figure 5E; Table S1). The RMC was then used to determine the dynamic range where quantification of the assay was deemed to be more reliable, i.e. concentration estimates corresponding to ≤2‐fold change in marker concentration. Ideally, the specific value for the RMC used to determine the dynamic range needs to be assessed via a separate clinical study outside the scope of this project.

The multiplexed assay was then tested using patients and “healthy” control samples (where samples were obtained from individuals without known disease) in both neat and diluted (1:100) forms. Each sample was tested three times across three days and day‐to‐day variation was found to be negligible (see Appendix S4). Assays which showed an increase in test line intensity signal upon dilution were regarded as impacted by the hook effect, and only the results of the diluted sample was therefore considered (see Appendix S5). In all other cases, only the undiluted patient samples were considered. Once all serum samples were tested with the optimized LFIA and test line intensities obtained, biomarker concentration values were extrapolated from the standard calibration curves (Figure 5A–C), which were fit using the heteroscedastic GP model. Assay sensitivity and specificity were then determined by plotting confusion matrices (as outlined in the Experimental Section, Table 5) where assay sensitivity and specificity for AFP was determined to be 87.5% and 98.3% and for HCG 100% and 100%, respectively, when compared to the gold standard measurements relative to their clinical cut off values (Table 2; Figure S12).

**TABLE 2:** Sensitivity, specificity, positive and negative predictive values (PPV and NPV) of each assay presented as percentages (see Table 5 for definitions).

Assay	AFP	HCG	CA125
Sensitivity (%)	87.5	100	10.0
Specificity (%)	98.3	100	95.7
PPV (%)	87.5	100	50.0
NPV (%)	98.3	100	71.4

Assay specificity was noted to be 95.7% for CA125, and the sensitivity 10%. Although this indicates good concordance between the LFIA test and gold standard measurements for AFP and HCG, it indicates little corroboration between the CA125 LFIA and the gold standard values assigned, with the LFIA demonstrating poor sensitivity. This appears surprising considering the promising LoD (4.61 IU/mL), which is markedly below the clinical cut‐off (35 IU/mL). However, we hypothesize that this is due to the vast heterogeneity of the CA125 protein (also known as mucin 16) and the absence of a universal CA125 standard used for assay calibration. The heterogeneity of CA125 has been largely attributed to extensive post‐translational modifications (namely, glycosylation) and degradation in the peripheral circulation [116, 117]. The variation in glycosylation subsequently affects the size of the marker, accounting for a large fraction of its mass, reaching the megadalton range and conformation of the protein, which could result in shielding one of its three main peptide epitopes (M11, OC125, and OV97) [118, 119]. This is evidenced by research conducted by the International Society of Oncology and Biomarkers demonstrating that different antibodies had varying success in detecting CA125 of different molecular weights, attributing this finding to steric effects [118]. Second, the type and degree to which the CA125 protein is glycosylated can vary between disease states, ovarian cancer cell lines, and subtypes with some subtypes such as high grade serous ovarian carcinoma (HGSOC) preferentially expressing CA125 with glycan forms such as sialyl‐Tn [116]. This heterogeneity, as a result of post‐translation modification, results in variable antibody interactions with overlapping CA125 tandem repeat domains [120, 121]. Further studies have also shown greater assay sensitivity in detecting the presence of HGSOC when specific glycosylation motifs were targeted as well as differences in the absolute CA125 concentration values assigned to the same patient samples when compared to commercial assays [122].

For these reasons, and unlike the AFP and HCG assays, no internationally agreed protein standard exists for CA125. CA125 protein standards developed for assay calibration are therefore manufacturer‐specific, and variations have been noted between manufacturers [123]. This is significant for multiple reasons: first, the anti‐CA125 antibodies selected and incorporated into the assay bind to peptide epitopes (M11 and OC125), which were optimized in the LFIA to bind to a specific commercial CA125 marker standard. This commercial standard was also used to determine the CA125 LFIA LoD value. However, the sensitivity of the assay was determined by comparing CA125 concentrations derived using the LFIA against those derived using the laboratory gold standard assay, the latter using its own internal standard. Given the well‐established heterogeneity of CA125, the use of different standards to benchmark the assays is a likely cause of discrepancy. Furthermore, the lack of an international CA125 standard and the subsequent use of a commercial protein standard for the LFIA optimization is not necessarily representative of the CA125 proteins found specifically in OGCT serum samples, with studies indicating varying levels of CA125 expression and epitope availability between different ovarian cancer subtypes [124]. Lastly, our findings appear to affirm, in the broader context of the literature, discrepancies noted in the performance of different commercial CA125 assays. Schröder et al. directly compared two CA125 immunoassays across patients with benign and malignant conditions, identifying significant variation in the correlation between immunoassay methods [125]. External quality assessment studies have also demonstrated significant discrepancies in CA125 detection across commercial assays, suggesting poor agreement across some clinically employed devices [123, 126]. Again, this discrepancy was attributed to the absence of standardized and certified reference materials, which would enable manufacturer benchmarking in a controlled manner, and a reliable comparison of quantitative marker concentrations across assays.

The exploration of CA125 forms expressed in OGCT alongside other ovarian cancer subtypes and identifying epitopes that are more representative of CA125 variations across relevant patient cohorts should form the basis of developing a commutable biomarker standard. This could then enable the generation of antibodies and other immunoglobulins raised against more specific epitopes, leading to more sensitive marker detection. It is important to note that the serial measurement of HCG and AFP alone is widely conducted in clinical practice without the addition of CA125 in the context of OGCT follow.up [14]. Although we included CA125 as a useful indicator of ovarian epithelial involvement, its absence from the multiplexed assay does not diminish the utility of the device for OGCT monitoring.

**FIGURE 6:**
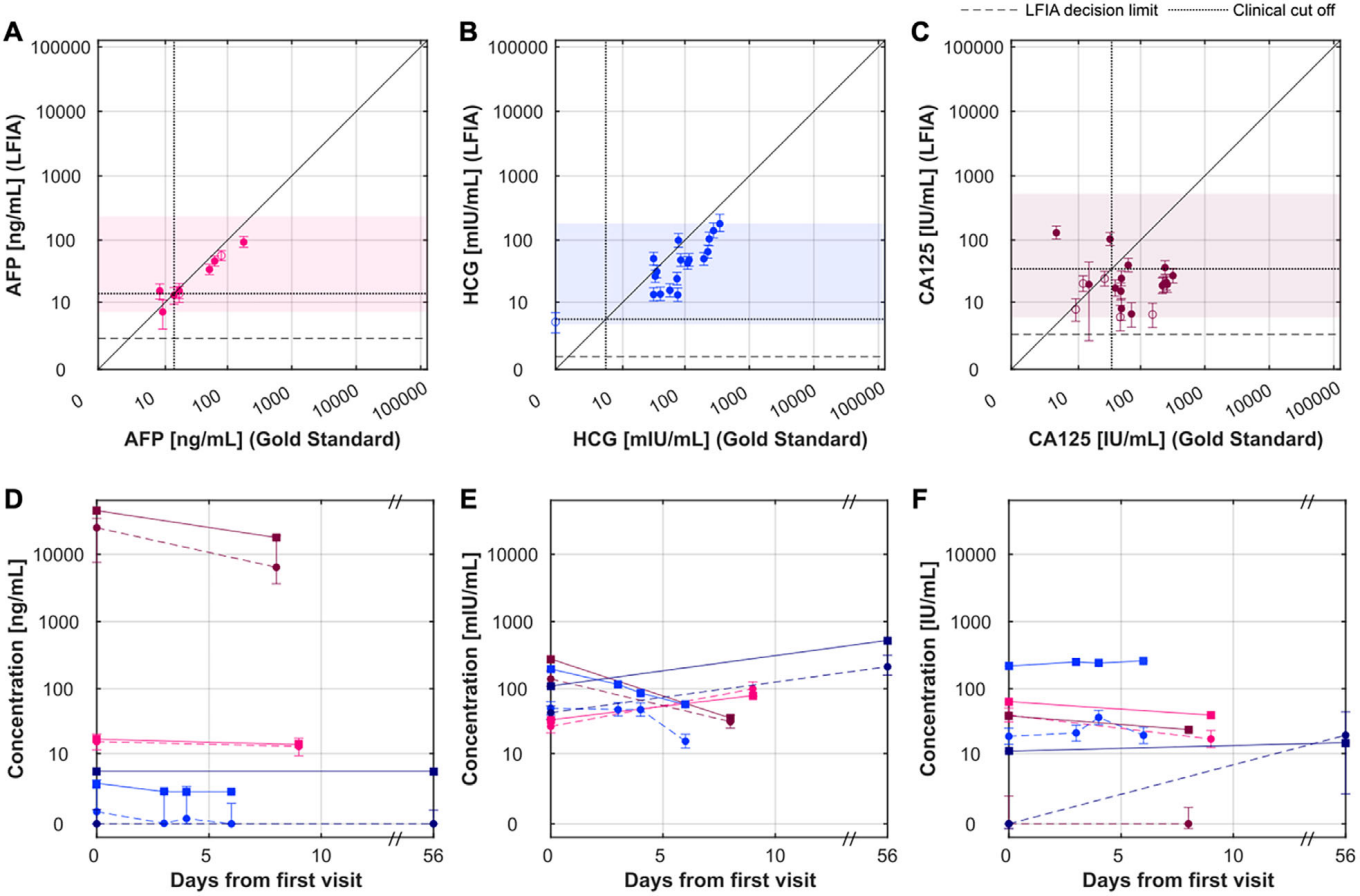
Graphs of A. AFP, B. HCG, and C. CA125 marker levels determined using the gold standard measurements plotted against the lateral flow immunoassays (LFIA) derived values falling within the dynamic range. The dynamic range is the shaded region of the graph. The LFIA concentration values were obtained by extrapolating signal test line values from the calibration curves in Figure 5. Longitudinal follow‐up of four patients testing D. AFP E. HCG, and F. CA125, comparing gold standard values (solid line and squares) and half‐dipstick LFIA values (dashed line and circles). Each color represents an individual patient. Data shown in panels A–F as mean ± 95% confidence interval, *N* = 3, *n* = 3, where *N* indicates the number of independent experiments conducted, including conjugation batches, and half‐dipstick assay preparation, and *n* refers to the number of half‐dipstick strips this was tested on at each marker concentration.

To estimate how marker concentration assays quantified by the LFIA device herein compared to the gold standard, for the AFP and HCG assays which showed high sensitivity and specificity, patient sample concentrations determined to be in the dynamic range as defined by the RMC (≤ 2) were subsequently plotted against their corresponding gold standard measurement and fitted with a linear regression model to estimate concordance (Figure 6A–B; Figure S11A–E). This demonstrated good concordance between the two assay modalities for AFP and HCG, as indicated by the gradient of the linear regression fit, equal to 0.93 and 0.84, respectively (Figure S11D,E). This was not conducted for CA125 due to the poor assay sensitivity observed (Table 2, Figure 6C).

Samples were also obtained for four patients who were monitored longitudinally using both the gold standard assay and the half‐dipstick assay developed herein. For both HCG and AFP, measurements obtained with the developed LFIA provided the same trends over time for each patient as compared with the gold standard assay (Figure 6D–F). This work indicates that the LFIA optimization and characterization pipeline developed herein can be applied to assays targeting different markers, showing good concordance when compared with laboratory gold standard assays in the presence of a universal marker standard.

## 3 Conclusions

Monitoring OGCT markers is critical for the early identification and management of the disease, which has a good prognosis when treated early, maximizing fertility preservation and minimizing health burden [9]. In this study, we develop a LFIA for the facile monitoring of OGCT markers. We present a robust and comprehensive method for optimization of the electrostatic conjugation of antibodies to peroxidase‐mimicking PtNZ probes using a DoE approach. Electrostatic conjugation is not only commonplace in diagnostics but also in therapeutics and many other fields. Careful optimization can enable the detection of markers within clinically relevant ranges without incurring additional costs associated with more complex bioconjugation strategies. Applying an I‐optimal DoE approach to probe conjugation optimization has revealed the presence of interactions between factors impacting signal generation and nonspecific binding at the test line, which a typical OFAT approach would not identify. Optimal conjugation parameters were shown to be antibody dependent, thus reaffirming the need for tailored, efficient, high‐throughput optimization strategies, made possible via DoE approaches. The RSM DoE models presented in this work were successfully validated, achieving clinically relevant LoDs with minimal further assay optimization. Additionally, we reviewed and adapted current methods for calculating LoDs that reflect the initial definition as reported by Currie et al., applying GPs to consider heteroscedasticity present across different marker concentrations in a way that has yet to be reported in the literature. Furthermore, we expanded upon the common metrics used to evaluate LFIAs for non‐communicable disease applications by including the RMC as an indicator of the assay's quantitative capabilities. The half‐dipstick assay was subsequently multiplexed and optimized, demonstrating clinically relevant LoDs and low RMCs in spiked pooled human serum with a minimum of 24 µL of serum required, enabling the use of a smaller lancet to maximize patient comfort. The optimized multiplexed assay has since shown to be largely concordant with gold standard laboratory benchtop assays when tested with patient samples benchmarked against an internationally recognized standard. To our current knowledge, the tri‐plexed, half‐dipstick LFIA presented in this work, incorporating catalytically active probes for the detection of OGCTs markers in serum, is the first of its kind in published literature.

## 4 Experimental Section

### 4.1 Materials Used for Nanoparticle Synthesis and Characterization

For Pt and Au nanoparticle synthesis 10 kDa polyvinylpyrrolidone (PVP) (Sigma‐Aldrich), chloroplatinic acid hydrate H_2_PtCl_6_ ≥ 99.9% purity (Sigma‐Aldrich), gold(III) chloride trihydrate HAuCl_4_·3H_2_O ≥ 99.9% purity (Sigma‐Aldrich), sodium citrate dihydrate ≥ 99.9% purity (Sigma‐Aldrich), L‐ascorbic acid 99% purity (Sigma‐Aldrich), and ultrapure distilled water (UPDW) DNAse/RNAse free (Invitrogen) were used. DLS UV‐Cuvette micro (Brand GMBH) and carbon‐coated 300 mesh copper grids (CF300‐CU50, Electron Microscopy Sciences) were used for characterization.

### 4.2 Buffers and Biological Material

Dulbecco's phosphate‐buffered saline (PBS) (Gibco) was used in any PBS‐based buffer or solution. For buffers of pH above 6.6, HEPES buffer was prepared using HEPES ≥ 99.5% purity (Sigma‐Aldrich) and UPDW. For buffers between pH 6 and 6.5 MES buffer was prepared using MES hydrate ≥ 99.5% purity (Sigma‐Aldrich) and UPDW. For buffers below pH 6 citrate buffer was prepared with sodium citrate dihydrate ≥ 99.9% purity (Sigma‐Aldrich) and citric acid ≥ 99.5% purity (Sigma‐Aldrich) in UPDW. Carbonate buffer (pH 9.6, 50 mm) for capture antibody coating of plate‐based assays was prepared with the addition of sodium carbonate (Sigma‐Aldrich) and sodium bicarbonate (Sigma‐Aldrich) in UPDW. Buffers were adjusted to desired pH using either 1 m NaOH prepared using NaOH ≥ 98% pellets (Sigma‐Aldrich) or 1 m HCl, using HCl 37% (Sigma‐Aldrich) in UPDW. Acetate buffer was prepared using sodium acetate (Sigma‐Aldrich) and acetic acid (Sigma‐Aldrich) in UPDW (pH 3.6, 0.1 m). Pooled normal human serum (Sigma‐Aldrich) was used as the LFIA sample matrix unless otherwise specified. Blocking agent solutions were all prepared in PBS and incorporated either 10 kDa PVP, β‐casein from bovine milk (Sigma‐Aldrich) or bovine serum albumin ≥ 96% lyophilized powder (Sigma‐Aldrich). Skimmed milk and fish gelatine, used in the sample and PtNZ diluents optimization were also purchased from Sigma‐Aldrich. PBST was prepared using Tween20 (Sigma‐Aldrich) at various concentrations as later indicated. Plate‐based colorimetric amplification solution was prepared in 50 mm citrate buffer at pH 5 and consisted of 0.01% w/v 3,3′,5,5′‐tetramethylbenzidine (TMB) in dimethylsulfoxide (DMSO) and 0.004% v/v H_2_O_2_. LFIA colorimetric amplification solution was prepared using Pierce CN/DAB Substrate Kit (Thermo Fisher Scientific) where a 1:9 of CN/DAB Solution (10×) and stable peroxide substrate solution (1×) were mixed in that order. All antibodies used were purchased from Medix Biochemica including anti‐AFP 105–15 (detection), anti‐AFP 105–16 (capture), anti‐HCG 5011 (detection), anti‐HCG 5014 (capture), anti‐CA125 4602 (detection), anti‐CA125 4601 (capture), except goat anti‐mouse ab13402 (control line) (Thermo Fisher Scientific). Please see Table S2 for Lot numbers. Protein markers purchased included: AFP recombinant antigen > 95% (LA443, Medix Biochemica), native HCG‐intact antigen > 95% (LA507L, Medix Biochemica), h CA125 (151‐25, Medix Biochemica), human CA125 calibrator grade (orB82215, Biorbyt), AFP WHO standard (72/225, NIBSC), Intact HCG sixth WHO international standard (18/244, NIBSC), Nicked HCG standard (99/692, NIBSC), and ß‐HCG standard (99/650, NIBSC).

### 4.3 Gold Nanoparticle Seed Synthesis

Citrate‐capped 15 nm gold nanoparticles (Au NPs) were synthesized by the addition of 10 mL of an aqueous solution of gold(III) chloride trihydrate (20 mm) to 180 mL of UPDW that was stirring at 100°C under reflux. The temperature was then decreased to 70°C and under vigorous stirring; the gold ions were reduced by the rapid addition of 10 mL sodium citrate dihydrate (68 mm) and incubated for 5 min. This was marked by the visible transformation of the reaction mixture to a deep red color. The ≈15 nm Au NPs solution was cooled down to room temperature and stored at 2–8°C. Sizes of Au seeds were characterized as per the nanoparticle characterization methods (see Section 4.5).

### 4.4 Platinum Core–Shell Nanoparticle Synthesis

PtNZs were synthesized using the method described by Loynachan et al. [45] In a typical synthesis, 120 nm PtNZs were produced by the addition of 31 µL (10 nm) of 15 nm Au NPs seeds to 969 µL UPDW followed by 10 kDa PVP in UPDW. PVP concentration was fine‐tuned initially using 40 µL of 20% w/v before selecting 20 µL of 20% w/v (the volume of UPDW was adjusted accordingly so final volume was 1100 µL, Figure s13). The solution was vortexed and incubated for at least 5 min before the addition of 40 µL of L‐ascorbic acid (100 mg/mL), followed by 40 µL of chloroplatinic acid (100 mm). This was then mixed and immediately incubated at 65°C for 30 min to allow for the reduction of the platinum ions on the gold seed surface. This is marked by the reaction mixture visibly turning black from a previously pale pink color. For larger synthesis volumes a magnetic stirer was added to ensure solution homogeniety. The PtNZs formed were then cooled to room temperature and excess reagents were washed off by centrifuging the solution four times at 1300 RCF for 10 min. The supernatant was removed and replaced with UPDW. The PtNZs were then characterized using DLS (see nanoparticle characterization methods below).

### 4.5 Nanoparticle Characterization

Size of nanoparticles were either determined by DLS using the Zetasizer nanoseries instrument (ZEN3600, Malvern Instruments) or by transmission electron microscopy (TEM) with the JEOL 2100F (Oxford Instruments) operating at 200 kV using the Gatan Orius SC 1000 SD camera equipped with the Gatan annular bright field and high‐angle annular dark field detectors. Elemental composition maps were obtained by energy dispersive x‐ray (EDX) spectroscopy in scanning transmission electron microscopy mode. For TEM analysis, samples were prepared by drop casting 2.5 µL of either 100 pm or 5 nm of PtNZs or Au NPs respectively onto carbon‐coated copper grids, which were left to dry for 1 h at room temperature before subsequent grid storage and sample imaging. For DLS, samples were prepared by diluting PtNZs to 10 pm in PBS and the instrument settings were fixed, using a 532 nm green laser and a 90° scattering detector angle.

### 4.6 Evaluation of PtNZ Catalytic Activity

The peroxidase‐mimicking activity of PtNZ was determined by assessing the colorimetric oxidation of TMB in the presence of H_2_O_2_ to determine the catalytic efficiency (*k*_cat_). The experiment was conducted in 0.1 m acetate (NaOAc/HOAc) buffer (pH 3.6) and absorbance was measured at 652 nm with the reported path length *l* = 0.588 cm. In a nonbinding 96‐well plate (Corning) 20 µL of 0.1 pm PtNZs was added to varying concentrations of TMB (1.6, 0.8, 0.4, 0.2, 0.1, 0.05, 0.025, and 0 mm —final concentration) and 2 m H_2_O_2_ (final concentration) to a final volume of 200 µL in acetate buffer. A negative control where MilliQ water was used instead of H_2_O_2_ was prepared for background subtraction. This was placed in the plate reader (Clariostar Plus) and monitored every 12 s over 15 min at room temperature. Changes in absorbance were plotted over time and the initial reaction rates were obtained from the linear portion of the plots (the first 36 s of the reaction). Initial reaction velocities (*ν*) were then calculated using the following equation: *ν* = (Δ*A*/Δ*t*)/(*ε* × *l*), where *A* is the change in absorbance over *t*‐time (min^−^^1^), *ε* is the absorbance coefficient of oxidized TMB (39,000 M^−1^·cm^−^^1^), and *l* is the pathlength which as previously mentioned was determined to be 0.588 cm. The relationship between initial velocities (*ν*, M·s^−^^1^) and TMB concentration was then modeled using the Michaelis–Menten equation: *ν* = *V*_max_ × *[S]*/(*K*_m_ + *[S]*), which was fitted using Prism 10.4.1 (GraphPad Software Inc) whereby *[S]* is the substrate or TMB concentration, *K*_m_ refers to the Michaelis constant, and *V*_max_ is the maximum velocity reached where *K*_m_ and *V*_max_ were derived using nonlinear regression. Catalytic efficiency was determined by calculating the *k*_cat_ value, which is determined by the following equation: *k*_cat_ = *V*_max_/*[E]*, where *[E]* refers to the final PtNZ concentration in the reaction (1 × 10^−14^ m). This experiment was repeated in the same manner but where H_2_O_2_ concentrations were varied (3.92, 1.96, 0.98, 0.49, 0.245, 0.1225, 0.06125, and 0 m—final concentration) and TMB concentration fixed (0.8 mm, final concentration) in a total volume of 200 µL in acetate buffer.

### 4.7 Antibody Conjugation to Platinum Nanozymes

Antibodies were conjugated to PtNZs using a protocol adapted from Loynachan et al. [45] whereby 10 µL of conjugating buffer was added to 100 µL PtNZs (300 pm), followed by 10 µL of antibody solution diluted in PBS. The solution was then vortexed and left to shake at 700 RPM at room temperature (20°C) for 3 h. 100 µL of blocking agent (either β‐casein, 10 kDa PVP or BSA) diluted in PBS was then added, the solution vortexed and then left to shake for 1 h at room temperature. For standard PtNZs conjugations used for plate‐based antibody validation assays, the conjugation buffer (HEPES) was fixed at pH 6.5 (100 mm) and blocking agent consisted of 2% w/v β‐casein in PBS and antibodies were diluted to an equivalent of 550 antibodies per PtNZ. Modified PtNZs were then washed three times to remove excess reagents by centrifugation at 1300 RCF for 10 min. The supernatant was replaced with a washing buffer consisting of 0.2% w/v of the blocking agent used in the conjugation in PBST containing 0.1% v/v Tween20. For the DoE conjugation optimization, the conditions varied as detailed in Table 1. The full list of DoE conjugation conditions for all three assays can be found in Figure S4A–C. The final optimized conjugation conditions for the multiplexed assay are as indicated in Table 3.

**TABLE 3:** List of final conjugation conditions for the three sandwich assays resulting from the DoE conjugation optimization process.

	Antibody equivalence (per PtNZ)	Blocking agent (wt%/vol%)	pH of buffer, ionic strength (mm)
AFP assay	830	β‐Casein, 2	6.26, 20
HCG assay	231	BSA, 0.2	4.5, 250
CA125 assay	100	BSA, 0.2	5.8, 250

### 4.8 Antibody Pair Validation in Plate‐Based Assays

To determine the signal‐to‐noise ratio of the sandwich assays, in a 96‐well high‐binding protein microtiter plate (Corning), 100 µL of 1 µg/mL solution of antibody diluted in 50 mm carbonate buffer (pH 9.6) was added per well, covered, and incubated at 4°C overnight. Post incubation, the plate was washed three times with 300 µL/well PBST (with 0.05% v/v Tween20) using the ELx405 HT automated microplate washer (BioTek). The appropriate antigen (AFP LA443 ‐ 100 µg/mL, HCG LA507L ‐ 110 mIU/mL, or CA125 151 ‐ 25–200 IU/mL) diluted in PBST (with 0.05% v/v Tween20) is added alongside a negative control (PBS) and incubated at room temperature (RT) for 30 min. The plate was washed again three times with PBST before 100 µL of the relevant PtNZ conjugate probes (1 pm, diluted in PBST with 0.05% v/v Tween20) were added and incubated at RT for another 30 min. After another wash step, the plate‐based colorimetric amplification buffer was added to each well and immediately covered preventing exposure to light. After 30 min 50 µL 4 m H_2_SO_4_ was added to stop further color development and absorbance was read at 450 nm using the Spectramax M5 plate reader (Molecular Devices).

To briefly optimize the capture and detection probe concentrations, the same protocol was employed as above but the capture antibody (anti‐AFP 105–16, anti‐HCG 5014, and anti‐CA125 4601) concentration was varied covering the concentration range of 0.0078–8 µg/mL across the length of the microtiter plate. Marker concentration was fixed for each assay and the respective functionalized PtNZ probes (conjugated with either anti‐AFP 105–15, anti‐HCG 5011, or anti‐CA125 4602) were also varied in concentration covering a range of 0.156–5 pm (10 pm for CA125) across the width of the microtiter plate effectively forming a checkerboard pattern. The rest of the assay was conducted as described above.

For the calibration curve assay the capture and detection antibodies were fixed in concentration as determined by the checkerboard assay with the capture antibody and probe conjugate concentrations being 1, 0.5, 0.5 µg/mL and 5, 2.5, 2.5 pm for the AFP, HCG, and CA125, respectively.

### 4.9 Constructing and Analyzing Design of Experiments Data

JMP pro 17 (SAS Institute) was used in the construction of the DoE table. A custom design was selected along with a response surface method interaction profile of the five conjugation parameters with an I‐optimality criterion. The five parameters included four 2‐level continuous variables and one 3‐level categorical variable. Center points were included and were repeated twice to give an indication of pure error. The number of experimental runs was minimized to 28 runs (Figure S4A–C). The design optimality criterion was set to I‐optimal to emphasize the generation of narrow confidence intervals for response predictions and the desirability criterion was set to maximize signal generation while minimize NSB, indicated as the two responses. Once experimental data were obtained, a model was fitted using stepwise least squares regression. Looking at the effect summary, model parameters were reduced till only significant parameters (*p* < 0.05) remained in the model (Figure S4D–F). Subsequently, accuracy of model fit and whole model statistical significance was established by the lack of fit and one‐way analysis of variation (ANOVA) tests, respectively. The prediction profiler was then plotted and set to maximum desirability to obtain the most optimal conjugation conditions for each assay (Figure S5). The maximization options to generate optimal conjugation conditions were set to 20 number of trips, 250 maximum iterations, 0.000001 convergence tolerance and 50 maximum cycles.

### 4.10 Preparation of Half‐Dipstick Assay

For the preparation of single test line assays, 1 mg/mL of capture antibody solution diluted in PBS was stripped onto CN95 nitrocellulose membranes (Unisart, Sartorious) using the Biojet automated liquid dispenser (BioDot) at 10 mm from the bottom of the strip at a rate of 1 µL/cm. The control line was stripped using goat anti‐mouse IgG (1 mg/mL) at 15 mm. For the final multiplexed LFIA, 1 mg/mL of anti‐CA125 4601 antibodies, anti‐HCG 5014, anti‐AFP 105–16 and goat anti‐mouse control antibodies were printed at 5, 8, 11, and 16 mm from the bottom of the CN95 nitrocellulose membrane respectively. The nitrocellulose membranes were subsequently dried in a 37°C oven overnight. This was then assembled onto an adhesive backing card (KN‐PS1060.44, Kenosha), and an absorbent pad (KN‐222‐20.1, Ahlstrom‐munksjo) was layered atop the nitrocellulose membrane with a 3 mm overlap. The adhesive sample pad region of the backing card was removed manually using scissors. The assembled membranes were then cut into 3 mm wide half‐dipstick strips using a guillotine cutter (CM5000, BioDot).

### 4.11 Testing DoE Conjugates in Half‐Dipstick Assay in Spiked Serum

15 µL of 100 pm conjugated PtNZs diluted in PBST (0.05% v/v Tween20) was added to 50 µL of a 4:1 mix of commercial human serum and a PBST (0.25% v/v Tween20) sample diluent spiked with antigen (or unspiked for control) in a 96‐well protein Loind microtiter plate (Corning). Final antigen concentrations were fixed at 14 ng/mL, 5 mIU/mL and 200 IU/mL for AFP, HCG, and CA125, respectively. For the DoE calibration curves the final antigen concentrations were 1134 ng/mL, 1215 mIU/Ml, and 12000 IU/mL of AFP, HCG, and CA125, respectively, which were serially diluted eight times by a factor of three. The respective half‐dipstick assays were then added to the mixture described above and incubated for 25 min, after which the assays were placed in the LFIA wash buffer (0.2% w/v β‐casein in PBST 0.1% v/v Tween20) for 10 min. The strips were then immersed into 600 µL of the CN/DAB amplification solution for 5 min and a final 30 s wash step in MilliQ H_2_O. Images of the assays were taken pre‐ and postamplification by placing strips on a grid using an iPhone 13 camera (Apple) in a mini studio lightbox to maintain lighting consistency.

### 4.12 Image Analysis of Half‐Dipstick Assay Strips

Once images were obtained, they were exported in jpeg format and analyzed in ImageJ (version 1.53k). Images were transformed into 8‐bit gray scale images and the gel analysis function was then used whereby rectangular regions with a 1:7‐1:8 width:height ratio were selected around the test lines and a region of the standardized grid. Pixel intensity profile plots of the selected regions were then generated. The peak raw pixel intensities for each assay test line (and the grid) were then exported and normalized by dividing the raw test line intensity with grid line intensity.

### 4.13 Testing Multiplexed Assay Cross‐Reactivity

The AFP, HCG, and CA125 capture antibodies were printed at 5, 10, and 15 mm, respectively, from the bottom of the assay in the initial iteration of the multiplexed LFIA. 15 µL of all conjugates at a final concentration of 100 pm each was added to 50 µL of the 4:1 mix of commercial human serum and sample diluent. The sample diluent was either spiked with AFP, HCG, CA125, all three markers or unspiked (as a control). The dipstick LFIA assay was incubated in the mixture and the remaining steps conducted as previously mentioned.

### 4.14 Optimization of Half‐Dipstick Assay

For optimization of probe concentration, a range of the DoE optimized conjugate concentrations were tested (100, 75, 50, 25, 12.5 pm) in the corresponding single‐plex assays (i.e., only one test line was printed at 10 mm height). All other steps remained consistent. For optimization of the test line height, three assays were fabricated where the capture antibodies against the three target markers were stripped onto the nitrocellulose at different heights as outlined in Table 4. 15 µL of a mixture of the optimized conjugates (100 pm anti‐CA125 PtNZs, 50 pm anti‐AFP PtNZs, and 50 pm anti‐HCG PtNZs) were added to 50 µL of the 4:1 serially diluted spiked serum in PBST outlined previously, and the rest of the assay was conducted as aforementioned for each assay test line variation.

**TABLE 4:** Three different multiplexed assay configurations where the three capture antibodies were printed at different heights.

Test line height from the bottom of the membrane	Assay variation 1	Assay variation 2	Assay variation 3
15 mm	CA125	AFP	HCG
10 mm	HCG	CA125	AFP
5 mm	AFP	HCG	CA125

The optimization of the composition of the sample and PtNZs diluent was conducted by screening different concentrations of Tween20 (1, 0.75, 0.5, 0.25, and 0.1% v/v) in the sample diluent and different types of blocking agents (0.5% w/v of either BSA, ß‐casein, PVP 10 kDa, and skimmed milk or fish gelatine in PBST) in both the sample and PtNZs diluents. The rest of the assay was carried out as previously described. To determine the minimum serum sample volume required, different volumes (40, 32, 24, 16 or 8 µL) of spiked and unspiked serum were mixed with the sample diluent in a 4:1 manner. After which the assay was submerged in the diluted serum and conducted as aforementioned. Throughout the optimization process the following commercial marker standards were used: AFP LA443, HCG‐intact LA507L, and CA125 or B82215.

### 4.15 Testing of the Optimized Half Dipstick Assay in Spiked Serum

In the final assay, 40 µL of a 4:1 mix of spiked serum sample and sample diluent (0.5% w/v β‐casein and 0.75% v/v Tween20 in PBS) was added to a 96‐well protein lo‐bind microtiter plate followed by 15 µL of the following PtNZs conjugate mix: 20 pm of anti‐HCG 5014, 25 pm of anti‐CA125 4602, and 50 pm of anti‐AFP 105–15 PtNZs. The PtNZs diluent consisted of 0.5% w/v β‐casein and 0.05% v/v Tween20 in PBS. The spiked serum samples were serially diluted by a factor of three covering the concentration ranges 0.519–3402 ng/mL, 0.185–1215 mIU/mL, and 1.296–8505 IU/mL for AFP, HCG, and CA125 markers, respectively. The WHO standards were used for the final assays (AFP‐72/225, HCG‐18/244) except for CA125 (orB82215). For the hook effect test, the maximum calibration concentrations were 81,000 ng/mL—AFP, 1,000,000 mIU/mL—HCG, and 20,000 IU/mL—CA125. The half dipstick assay was submerged in the sample/PtNZs solution for 20 min followed by the wash step incubation in 100 µL of wash buffer (0.2% w/v β‐casein in PBST 1% v/v Tween20) for 10 min. This is then followed by the 5‐min amplification step where the half‐dipstick assay is submerged in 600 µL of 9:1 CN/DAB:H_2_O_2_ followed by a quick wash step in purified MiliQ H_2_O for 30 s. Pre‐ and postamplification images were taken as described above.

### 4.16 Clinical Validation of Multiplexed Assay

For the clinical validation of the optimized assay, thirty‐one GCT patient serum samples were obtained for this work (reference number R23043) from the gestational trophoblastic disease biobank with permission of the Imperial College Healthcare Tissue Bank (ICHTB) under the subcollection reference number ONC_RH_13_051. The ICHTB is approved by Wales REC3 to provide human material for research (22/WA/0214). Thirty‐nine “healthy” serum samples were subsequently obtained from the commercial tissue bank, Central Biohub. All serum samples were received frozen and were thawed on the day of use under mild agitation. For each sample 32 µL of serum was diluted with 8 µL of the optimized sample diluent. To screen for hook effect a further 1 in 100 dilution was conducted where the neat sample was added to the hook effect diluent consisting of 0.2% w/v β‐casein, 0.1% v/v Tween20 in PBS. 32 µL of the diluted sample was also mixed with 8 µL of the sample diluent after which the optimized assay was tested as mentioned previously. The clinical validation took place over three days whereby three independent experimental repeats were conducted each day. Preceding the serum sample tests, full calibration tests were conducted each day. Patient samples number 4 and 21 were eventually omitted from the analysis due to increased turbidity and viscosity of the serum.

### 4.17 Statistical Analysis

All computations relating test‐line intensities to antigen concentrations and RMC calculations were performed in MATLAB R2022a Update4 (9.12.0.2009381; MathWorks Inc., Natick, MA, USA) using the Statistics and Machine Learning Toolbox and the Optimization Toolbox. The custom MATLAB function GP_heteroscedasticity (v2.0.0), which implements heteroscedastic Gaussian Process regression with automatic noise‐variance modeling, is publicly available on GitHub at https://github.com/ceeskildsen/GP_heteroscedasticity and archived on Zenodo [127]. LoD and *L*_C_ were estimated with a GP‐based probability‐rule bootstrap (*B*  =  1000), using per‐level parametric/residual resampling, standard deviation shrinkage toward a pooled low‐end standard deviation, and a blank standard deviation floor. For homoscedastic calculations a MATLAB toolbox developed by Miller et al. was used as described in their work [91]. This is available from: https://github.com/bensmiller/detection‐limit‐fitting. JMP v.17.2 (SAS) was exclusively used for the DoE construction and analysis. Statistical tests comparing efficacy of blocking agents and Tween20 concentration were conducted in Prism 10.4.1 (GraphPad Software Inc) following normality tests. Linear regression of validation samples and Michaelis–Menten plots were also conducted in Prism. Checkerboard assays were fitted in Prism with the two sites‐specific binding package. Two‐way ANOVA was conducted in Prism to compare the significance of the effects of the different assay conditions in Figure S9D–F, where significance was set as *p* < 0.05. All graphs depicted are plotted in Prism except for Figures 5 and 6 and Figure S11A–C. For specifics on calibration curve modeling and LoD calculations please see Appendixes S2 and S3. Most experiments were either conducted as *N* = 3, *n* = 3 or *N* = 1, *n* = 3 replicates, where *N* indicates experimental replicates and *n* indicates technical replicates unless otherwise specified in the figure legend.

### 4.18 Resolution of Molecular Concentration (RMC)

The RMC is derived from the calibration models, following the definitions and methods outlined by Wilson et al. [42] The RMC represents the smallest fold change in analyte concentration that can be statistically distinguished at a given concentration level. In this study, we calculate the RMC using a 95% confidence interval, incorporating heteroscedasticity to account for variations in measurement noise across different concentration levels.

### 4.19 Assay Sensitivity, Specificity, and Coefficient of Variation

The coefficient of variation of an assay is calculated by dividing the standard deviation of test line signal intensities at a given antigen concentration by the mean test line intensity. This value is then averaged across all antigen concentrations tested and multiplied by one hundred to obtain a percentage value.

Assay sensitivity, specificity, positive and negative prediction values (PPV and NPV) are described in relation to their clinical cut‐offs and the gold standard measurements. First, a confusion matrix is plotted (Table 5) whereby values above the clinical cut‐off as identified by both the gold‐standard and LFIA dipstick are considered true positive with the opposite being considered true negative. Therefore, sensitivity is defined as the ability of the multiplexed LFIA to detect antigen concentration in samples that are above the clinical cut‐off as deemed by the gold standard method. Specificity is defined as the number of samples determined to be antigen negative (or below the cut‐off) as compared to those identified by the gold standard measurements. We refrained from classifying by disease status as biomarker concentration is but one indicator of disease status that must be coupled with further unobtainable patient information.

**TABLE 5:** Confusion matrix detailing sensitivity, specificity, positive and negative predictive value (PPV and NPV) calculations.

		Gold standard values		
		Positive	Negative	
LFIA values	Positive	A—True positive	B—False positive	PPV: [A/(A + B)] × 100
	Negative	C—False negative	D—True negative	NPV: [D/(D + C)] × 100
		Sensitivity: [A/(A + C)] × 100	Specificity: [D/(D + B)] × 100	

### 4.20 Ethics Approval Statement

Human samples used in this research project were obtained from the Imperial College Healthcare Tissue & Biobank (ICHTB). ICHTB is supported by the National Institute for Health Research (NIHR) Biomedical Research Centre based at Imperial College Healthcare NHS Trust and Imperial College London. ICHTB is approved by Wales REC3 to release human material for research (22/WA/0214) and the samples for this project (R23043) were issued from subcollection reference number ONC_RH_13_051.

### 4.21 Human Research Participants

All commercial distributors of human serum samples used within this study have confirmed to have obtained written consent from participants in the first instance that these samples were taken.

## Author Contributions

A.A. was responsible for most of the work, including conducting plate‐based and lateral flow immunoassays, nanozyme synthesis, characterization, and conjugation as well as some of the statistical analysis. C.E.E. conducted heteroscedastic LoD, RMC, and patient validation statistical analysis. C.J.S. conducted some PtNZ conjugations and nanozyme peroxidase activity characterization. C.J.S. and Y.C. conducted TEM sample preparation and imaging of the nanoparticles. S.C. and C.J.S. advised and supported establishing the LFIA developmental milestones and characterization. F.G. advised and supported DoE experimentation. S.S. and E.H.W. provided advice and oversight over obtaining patient serum samples and the clinical validation process. L.P. and A.S. provided supervision, oversight and support for the synthesis and DoE led conjugation of the nanozymes as well as assay optimization and validation. A.A. drafted the paper, while C.E.E., L.P., A.S., F.G., E.H.W., Y.C., C.J.S., S.C., S.S., and M.M.S. all revised the paper. S.S. and M.M.S. supervised the study.

## Funding

A.A., S.S., and M.M.S. acknowledge funding from Cancer Research UK (C309/A31316) and the Rosetrees Trust. L.P. acknowledges funding from the European Union's Horizon Europe research and innovation programme under the Marie Skłodowska‐Curie Actions (101106805). C.E.E. acknowledges funding from the Engineering and Physical Sciences Research Council (EPSRC) under the UK Research and Innovation (UKRI) through the project “Trustworthy Decision Limits for Multiplexed Diagnostics” (Trust‐MDx; EP/Y023498/1). A.S. and M.M.S. acknowledge funding from the EPSRC IRC Agile Early Warning Sensing Systems for Infectious Diseases and Antimicrobial Resistance (EP/K031953/1 and EP/R00529X/1). Y.C. acknowledges funding from the President's Ph.D. Scholarship at Imperial College London. S.S.C. acknowledges support from Fundação para Ciência e Tecnologia (SFRH/BD/147881/2019). M.M.S. acknowledges funding from the Department of Science, Innovation and Technology (DSIT), the Royal Academy of Engineering under the Chair in Emerging Technologies programme (CiET2021∖94), and the University of Oxford Strategic Research Fund.

## Conflicts of Interest

M.M.S. has invested in, consults for (or is on scientific advisory boards or boards of directors) and conducts sponsored research funded by companies related to the biosensing field; has filed patent applications related to biomaterials; and has co‐founded companies in the biomaterials field. C.J.S., S.C., and A.S. have consulted for a company related to nanomaterials and assays for biosensing. C.J.S. and A.S. have filed patent applications relating to nanomaterials for biosensing. The rest of the authors declare no conflict of interest.

## Supporting information




**Supporting File**: advs74220‐sup‐0001‐SuppMat.docx.

## Data Availability

Raw research data is available online at DOI: 10.5281/zenodo.18557825.
