# Supplementary material for: A Rational Optimization Approach for the Development of a Multiplexed Lateral Flow Immunoassay: Detection of Nonepithelial Ovarian Cancer Markers in Human Serum
Source: Adv Sci (Weinh). 2026 Feb 25;13(31):e23192. doi: 10.1002/advs.202523192 (PMC13587956; doi:10.1002/advs.202523192)
Supplement: Supplementary file 1 [file ADVS-13-e23192-s001.docx]

## A Rational Optimisation Approach for The Development of a Multiplexed Lateral Flow Immunoassay Detecting Non-Epithelial Ovarian Cancer Markers in Serum

*Aida Abdelwahed, Carl E. Eskildsen, Luca Panariello, André Shamsabadi, Christy J. Sadler, Edmund H. Wilkes, Federico Galvanin, Yuxi Cheng, Sara Carvalho, Srdjan Saso, Molly M. Stevens**

**A. Abdelwahed, C.E. Eskildsen, L. Panariello, A. Shamsabadi, C.J. Sadler, Y. Cheng, S. Carvalho, M.M. Stevens**

Department of Materials, Department of Bioengineering and Institute of Biomedical Engineering, Imperial College London, London, SW7 2AZ, U.K.

Corresponding author E-mail: molly.stevens@dpag.ox.ac.uk

**C.E. Eskildsen, L. Panariello, A. Shamsabadi, C.J. Sadler, Y. Cheng, M.M. Stevens**

Department of Physiology, Anatomy and Genetics, Department of Engineering Science, and Kavli Institute for Nanoscience Discovery, University of Oxford, Oxford, OX1 3QU, U.K.

**L. Panariello, M.M. Stevens**

Department of Medical Biochemistry and Biophysics, Karolinska Institutet, Stockholm, SE-171 77, Sweden

**E.H. Wilkes**

Department of Clinical Biochemistry, NorthWest London Pathology, Imperial College Healthcare NHS Trust, Charing Cross Hospital, London, W6 8RF, U.K.

**F. Galvanin**

Department of Chemical Engineering, University College London, London, WC1E 7JE, U.K.

**S. Saso**

West London Gynaecological Cancer Centre, Hammersmith Hospital, Imperial College NHS Trust, London, W12 0HS, UK.

**S. Saso**

Department of Metabolism, Digestion and Reproduction, Imperial College London, London, W12 0NN, U.K.

**Figure S1. A.** Signal and noise values for different antibody combinations for the three assays AFP, HCG and CA125. Data shown as mean N=1, n = 3. **B.** Signal-to-noise ratio of the three assays switching antibody capture and detection antibodies. **C.** AFP, **D.** HCG and **E.** CA125 checkerboard assays to optimise the concentration of detection probes and capture antibodies for the plate based calibration curve for the **F.** AFP, **G.** HCG and **H.** CA125 assays and **I.** table of corresponding numerical values. Calibration curve data shown as mean ± S.D. of N=1, n = 3. N indicates the number of independent experiments conducted; n refers to the number of NLISA wells used at each marker concentration. Cap- refers to the capture antibody, Det- refers to the detection antibody.

**Figure S2.** Characterisation of PtNZ catalytic activity. **A.** Time-resolved evolution of the absorbance at 652 nm caused by the oxidation of different concentrations of TMB (mM) in the presence of fixed H_2_O_2_ (2 M) concentration catalyzed by PtNZs. **B.** Plots depicting dependance of initial reaction velocity (v, M.s^-1^) obtained from plot **A** in the first 36 seconds of the reaction on TMB concentration. **C.** Time-resolved evolution of the absorbance at 652 nm caused by the oxidation of different concentrations of H_2_O_2_ (M) in the presence of fixed TMB concentration (0.8 mM) catalyzed by PtNZs. **D.** Plots depicting dependance of initial reaction velocity (v, M.s^-1^) obtained from plot **C** in the first 36 seconds of the reaction on H_2_O_2_ concentration. Data shown as mean ± SD of n = 3 separate plate wells. Vmax and Km were determined using the Michaelis-Menten fitting of B and D after which the catalytic efficiency (k_cat_) was calculated and plotted in table **E** in comparison with the enzyme horseradish peroxidase (HRP). [E] represents the enzyme concentration used. HRP values were obtained from the literature^1^.

**Figure S3. A**. Dynamic light scattering (DLS) intensity size distribution of bare platinum nanozymes (PtNZs) and antibody conjugated PtNZs. **B.** Conjugation mixture of anti-CA125 antibodies with PtNZs incubated under acidic conditions where aggregation is clearly visible with pellets forming, leaving a lighter supernatant at lower pH. DLS data of all the conjugates used in the **C.** AFP, **D.** HCG and **E.** CA125 design of experiments depicting the polydispersity index values for all PtNZ conjugated which is less than 0.15 (dashed line) and Z-average diameter (nm). Data depicts n=3 mean ± SD for each DLS measurement repeat **F.** Electrostatic conjugation parameters incorporated in the design of experiments models.

**Figure S4A-C.** List of 28 conjugation conditions as determined by the custom design in JMP for each of the three assays **A.** AFP, **B.** HCG and **C.** CA125 in the design of experiments (DoE) approach to optimisation.

**Figure S4D-F.** Model parameters **i.** before removal of insignificant parameters and **ii.** after stepwise regression retaining only significant parameters for the **D.** AFP, **E.** HCG and **F.** CA125 assays. ^ denotes parameters that are insignificant, but which are contained in significant model parameter interactions.

**Figure S4G.** Full model expressions for **i.** signal and **ii.** non-specific binding (NSB) for the AFP assay.

**Supplementary Figure S4H.** Full model expressions for **i.** signal and **ii.** non-specific binding (NSB) for the HCG assays.

**Figure S4I.** Full model expressions for **i.** signal and **ii.** non-specific binding (NSB) for the CA125 assay.

**Figure S5A-C.** Prediction profilers of conjugation conditions when the desirability criteria are set for maximising signal generation and minimising NSB for the three assays **A.** AFP, **B.** HCG and **C.** CA125 assays.

**Figure S6.** Prediction profilers plotted using the DoE models assessing the impact of the interaction of pH **Ai.** acidic or **Aii.** alkaline and antibody equivalence on signal and non-specific binding (NSB) in the HCG assay. Prediction profiler was used to assess the interaction between blocking agent and conjugation buffer pH in the **Bi, Bii** AFP and **Ci, Cii** CA125 assays. It was also used to assess the interaction between the blocking agent **Di.** PVP or **Dii.** ß-casein and antibody equivalence in the AFP assay.

**Figure S7**. Results of the first iteration of the multiplexed assay tested with spiked human serum at different antigen concentrations mixed with the design of experiments optimised conjugates and corresponding calibration curves for **A.** CA125, **B.** HCG and **C.** AFP assays. Data shown as mean ± S.D. N=1, n = 3. N indicates the number of independent experiments conducted, including conjugation batches and half-dipstick assay preparation; n refers to the number of half-dipstick strips used to test samples at each marker concentration. **D.** Image of multiplexed assay tested with serially diluted antigen. **E.** Numerical values and limits of detection corresponding to graphs A-C.

**Figure S8. A.** Effect of test line distance from bottom of nitrocellulose membrane on the **i.** CA125 assay performance **ii,iii** HCG assay performance and **iv,v** AFP assay performance. **B.** Effect of PtNZ conjugate concentration on the LoDs of the **i.** CA125, **ii,iii** HCG and **iv,v** AFP assays. **C.** The impact of extremely high marker concentration on assay performance for **i.** CA125 and **ii.** AFP detection. Data shown as mean ± S.D. of N=1, n = 3. N indicates the number of independent experiments conducted, including conjugation batches and half-dipstick assay preparation; n refers to the number of half-dipstick strips used to test samples at each marker concentration. Data in Ai and Bi correspond to graphs in figures 4C and 4B respectively.

**Figure S9.** **A.** Effect of surfactant concentration in the sample diluent on test line intensity at different **A.** AFP, **B.** HCG and **C.** CA125 marker concentrations. **D.** Impact of incorporating β-casein in the sample diluent, PtNZs diluent or in both on test line intensity in comparison to a negative control. **E.** Screen of different agents to incorporate into diluent buffer and their impact on test line intensity across the three assays**. F.** Assessing the impact of different serum sample volumes on assay performance to determine the minimum volume required before assay function is impaired. Two-way ANOVA was conducted to compare the significance of the effects of the different assay conditions, * indicates significance p< 0.05, ** p<0.01, *** p<0.001, ****p<0.0001. Data shown as mean ± S.D. of N=1, n = 3. N indicates the number of independent experiments conducted, including conjugation batches and half-dipstick assay preparation; n refers to the number of half-dipstick strips used to test samples at each marker concentration.

**Figure S10.** Impact of two different incubation times on test line intensity of the **A.** AFP assay, **B.** HCG assay and **C.** CA125 assay. The 35-minute total assay time comprised of 20 minutes incubation in serum and platinum nanozyme (PtNZ) probes, 10 minutes incubation in wash buffer and 5 minutes incubation in amplification buffer. The 20-minute assay time comprised of 10 minutes in sample and PtNZs, 5 minutes in wash buffer followed by 5 minutes in amplification buffer. Data shown as mean ± S.D. of N=1, n = 3. N indicates the number of independent experiments conducted, including conjugation batches and half-dipstick assay preparation; n refers to the number of half-dipstick strips used to test samples at each marker concentration.

**Table S1.** Numerical parameters for optimised multiplexed calibration curve (in Figure 5A-C) including the critical limit (Lc), IC10 (concentration corresponding to 10% of total assay signal), limit of detection (LoD), smallest resolution molecular concentration fold change (μ_min_) and the concentration at the μ_min._

| **Calibration Curve Parameter** | **AFP**  **ng/mL** | **HCG**  **mIU/mL** | **CA125**  **IU/mL** |
| --- | --- | --- | --- |
| L_C_ (norm. intensity) | 0.13 | 0.08 | 0.13 |
| IC10 | 8.80 | 5.14 | 8.76 |
| Homoscedastic LoD | 6.30 | 2.70 | 7.48 |
| Heteroscedastic LoD | 5.11 | 1.55 | 4.61 |
| Hill Slope | 1.10 | 0.91 | 0.88 |
| μ_min_ | 1.36 | 1.55 | 1.43 |
| Concentration at μ_min_ | 53.0 | 37.1 | 89.2 |

**Figure S11.** Graphs of **A.** AFP, **B.** HCG, **C.** CA125 marker levels determined using the gold standard measurements plotted against the lateral flow immunoassays (LFIA) derived values. Outliers (due to sample turbidity) highlighted in green. Shaded region corresponds to the RMC determined dynamic range. Linear regression plots of **D.** AFP and **E.** HCG marker levels determined using the gold standard measurements plotted against the lateral flow immunoassays (LFIA) derived value falling within the dynamic range. Data has been log transformed. Data shown as mean ± 95% confidence interval of N=3, n = 3. N indicates the number of independent experiments conducted, including conjugation batches and half-dipstick assay preparation; n refers to the number of half-dipstick strips used to test samples at each marker concentration.

**Figure S12.** Confusion matrices plotted after testing patient serum samples and comparing to gold standard values for the **A.** AFP, **B.** HCG and **C.** CA125 assays.

**Figure S13.** Transmission Electron Microscopy (TEM) images of **A.** Platinum Nanozymes (PtNZs) synthesised with 20 µL 20% w/v 10kDa PVP and **B.** Images of 15 nm Au nanoparticles synthesised as the core of the PtNZ.

**Table S2.** List of antibodies used in experimentation including lot numbers.

| **Antibody** | **Catalogue number.** | **Lot. Numbers** |
| --- | --- | --- |
| Anti-AFP 105-15 | 105-15 | 05H6031 |
| Anti-AFP 105-16 | 105-16 | 03H6987 |
| Anti-HCG 5011 | 100009 | 2002120 |
| Anti-HCG 5014 | 100011 | 2001377, 2001984 |
| Anti-CA125 4601 | 100598 | 2002168, 2003572 |
| Anti-CA125 4602 | 100628 | 2001988, 2002463 |
| Anti-mouse | ab13403 | GR3373526-2 |

***Appendix 1***

To calculate the number of antibodies needed to form a monolayer, the PtNZs were assumed to be a perfect sphere with a radius of 60 nm and the average cross sectional length of an IgG antibody was calculated to be 9 nm, based on the dimensions of a typical IgG antibody reported in previous work.^2,3^ Each of the three antibody faces were added and divided by three assuming an even split in antibody orientation on the particle surface:

$$\frac{14.5 nm+8.5 nm+4 nm}{3}=9 nm$$

The maximum antibody loading onto the PtNZ surface (radius of 60 nm) with a mixture of orientations having calculated an approximate surface area was therefore:

$$\frac{Area of PtNC}{Area of antibody} = \frac{{4\pi(60\times{10}^{-9})}^{2}}{81\times{10}^{-18}} =559 Ab/PtNC \left( Eq.1 \right)$$

***Appendix 2 – Homoscedastic modelling of assay calibration curves***

Homoscedastic calibration curve models were conducted as outlined by Holstein *et al.* in comparison to the heteroscedastic models presented herein ^4^. For this a four-parameter regression model was fitted to the graphs as outlined below:

𝑦=I𝑚𝑖𝑛+(I𝑚𝑎𝑥−I𝑚𝑖𝑛)/(1+(EC50/log𝑥)^𝐻𝑖𝑙𝑙𝑆𝑙𝑜𝑝𝑒))	$\left( Eq.2 \right)$

Where Imin is the minimum test line intensity and Imax is the maximum test line intensity, the EC50 is the antigen concentration corresponding to 50% of the maximal test line intensity achieved. The hillslope is the gradient of the linear region after the first inflection point of the sigmoidal curve. The LoD was then calculated using the Holstein *et al.* method in a MATLAB toolbox developed by Miller *et al.* as referenced in their work.^4–6^

***Appendix 3 - Heteroscedastic modelling of assay calibration curves using Gaussian processes regression***

As a kernel function, we use the Radial Basis Function (RBF), also known as the squared exponential kernel. It is a widely used kernel in GP regression due to its flexibility and smoothness properties.^7^ It assumes that points closer together in the concentration domain (i.e., input space) are more strongly correlated in the signal domain (i.e. output space), with the strength of the correlation decreasing as the distance between points in the concentration domain increases. Mathematically, the RBF kernel is defined as:

$$\begin{aligned} k\left( x_{i},x_{j} \right)=\sigma^{2}\exp\left( -\frac{\left( x_{i}-x_{j} \right)^{2}}{2l^{2}} \right)\#\left( Eq.3 \right) \end{aligned}$$

where $x_{i}$ and $x_{j}$ corresponds to the analyte concentration of samples *i* and *j*, respectively, $\sigma^{2}$ is the signal variance, which controls the scale of the correlation between points in the input space, and *l* is the length scale, determining how quickly the correlation decays with distance. Both *σ* and *l* are hyperparameters of the GP model, and their values are estimated from the training data by minimizing the negative log marginal likelihood. This process optimizes the hyperparameters to best explain the observed data while balancing model complexity and data fit.

The predictions of new samples, given the training data *D*, are provided by the mean function, $\boldsymbol{\mu}_{y_{*}|D}$,

$$\begin{aligned} \boldsymbol{\mu}_{\boldsymbol{y}_{\boldsymbol{*}}\boldsymbol{|D}}\boldsymbol{=}\boldsymbol{K}_{\boldsymbol{*}}^{\boldsymbol{T}}\left( \boldsymbol{K+I}\boldsymbol{\sigma}_{\boldsymbol{n}}^{\boldsymbol{2}} \right)^{\boldsymbol{-1}}\boldsymbol{y} \#\left( Eq. 4 \right) \end{aligned}$$

where ***K*** is the covariance matrix of the training data, $\boldsymbol{\sigma}_{n}^{2}$ is the noise variance (a vector as noise varies across the concentration domain), ***I*** is the identity matrix, ***y*** is a vector of observed test line intensities, and $\boldsymbol{K}_{*}$ is the covariance between the training data and the new samples. The covariance matrices are estimated using the kernel function. To account for heteroscedasticity, the elements of $\boldsymbol{\sigma}_{n}^{2}$ are estimated as the variance between replicates at each concentration, allowing for varying levels of noise across different concentrations.

The variance at each new sample, given the training data *D*, is provided by,

$$\boldsymbol{\Sigma}_{y_{*}|D}=\boldsymbol{K}_{**}+\boldsymbol{I}\boldsymbol{\sigma}_{n*}^{2}-\boldsymbol{K}_{*}^{T}({\boldsymbol{K}+\boldsymbol{I}\boldsymbol{\sigma}_{n}^{2})}^{-1}\boldsymbol{K}_{*} (Eq.5)$$

where $\boldsymbol{K}_{**}$ is the covariance matrix of the new samples, estimated by the kernel function, and $\boldsymbol{\sigma}_{n*}^{2}$ contains the noise variance for each new sample, accounting for heteroscedasticity. To estimate the noise variance $\sigma_{n*}^{2}$ for each new sample, an auxiliary GP model is used. This secondary GP, employing the same RBF kernel, is trained on the training data with concentrations as the input space and the noise variances ($\boldsymbol{\sigma}_{n}^{2}$) of the training data as the output space. By modelling the noise variance as a function of concentration, the framework effectively captures heteroscedasticity in the data, enabling more accurate variance predictions for new samples.

**Bootstrap engine and LoD by probability rule.** For each bootstrap $b=1,\ldots,B$, we simulate responses at each observed concentration level using a Gaussian model with per-level means and standard deviations. To stabilise small-sample standard deviations we shrink per-level standard deviations toward a pooled low-concentration standard deviation ($\lambda=0.35$) and apply a numerical floor ($\varepsilon={10}^{-6}$). Blanks are re-simulated if their draws are degenerate (≤1 unique value, up to 20 respins). A heteroscedastic GP is re-fitted on the simulated data and the predictive distribution $Y\mid x$ gives $P\left( Y>L_{C} \mid x \right)$ where $L_{C}$ is the one-sided $t$ threshold from blanks at $\alpha=0.05$. The LoD is $min\{x:\text{ }P(Y>L_{C}\mid x)\geq0.95\}$. We summarise $L_{C}$ (signal units) and LoD (concentration) by the bootstrap mean. This couples Currie’s two-error LoD with heteroscedastic GP inference and propagates calibration uncertainty.

***Appendix 4 - Estimating the significance of day-to-day variation***

We assessed whether measurements exhibit a day effect after controlling for concentration. We fitted two heteroscedastic Gaussian-process (GP) models: a concentration-only model with an RBF kernel $\boldsymbol{K}_{\text{conc}}$(reduced), and a nested model that adds an additive day component. The full model is

$$\boldsymbol{K}_{\text{full}}\text{ }=\text{ }\boldsymbol{K}_{\text{conc}}\text{ }+\text{ }\sigma_{\text{day}}^{2}\text{ }\delta\left( \text{day}_{i}=\text{day}_{j} \right) (Eq.6)$$

The reduced model is recovered when $\sigma_{\text{day}}^{2}=0$. Per-input noise was estimated from replicate measurements at identical inputs. We compared models with a likelihood-ratio test (LTR; 1 degree of freedom; parameter on the boundary, so $\chi_{1}^{2}$is used conservatively) and also quantified practical impact via the variance share $\hat{\sigma}_{\text{day}}^{2}/Var(y)$(95% profile-likelihood CI) and leave-one-day-out cross-validated RMSE.

Although the LRT can detect a statistically non-zero day component in-sample, the estimated variance share is small and there is no out-of-sample improvement (ΔRMSE ≈ 0 or positive). We therefore treat day-to-day variation as not operationally significant and proceed with the concentration-only model.

***Appendix 5 - Predictions***

The test line intensities for both patient and control samples were averaged across the three days and analyte concentrations were estimated from these averaged values by extrapolating from the calibration curve models. Uncertainties in the estimated concentrations were also derived from the calibration models. For each sample, both diluted and non-diluted versions were analysed, and the concentration estimate yielding the highest value was selected as the final concentration estimate for the sample. This was conducted keeping the hook effect in mind where samples affected by this artefact would yield greater signal intensity upon dilution whereas those unaffected would yield higher signal intensity before dilution.

**References**

1.    Gao, L. *et al.* Intrinsic peroxidase-like activity of ferromagnetic nanoparticles. *Nat. Nanotechnol.* **2**, 577–583 (2007).

2.    Valentine, R. C. & Green, N. M. Electron microscopy of an antibody-hapten complex. *J. Mol. Biol.* **27**, 615–617 (1967).

3.    Silverton, E. W., Navia, M. A. & Davies, D. R. Three-dimensional structure of an intact human immunoglobulin. *Proc Natl Acad Sci USA* **74**, 5140–5144 (1977).

4.    Holstein, C. A., Griffin, M., Hong, J. & Sampson, P. D. Statistical method for determining and comparing limits of detection of bioassays. *Anal. Chem.* **87**, 9795–9801 (2015).

5.    Miller, B. S. *et al.* Sub-picomolar lateral flow antigen detection with two-wavelength imaging of composite nanoparticles. *Biosens. Bioelectron.* **207**, 114133 (2022).

6.    Miller, B. S. Detection Limit Fitting Tool. https://github.com/bensmiller/detection-limit-fitting.

7.    Rasmussen, C. E. & Williams, C. K. I. *Gaussian processes for machine learning*. (The MIT Press, 2006). doi:10.7551/mitpress/3206.001.0001.
